# Investigating the therapeutic mechanism of Bufei Decoction in COPD: Schisandrin B targets the TLR4/NF-κB/JAK-STAT signaling pathway

**DOI:** 10.1186/s41065-025-00629-8

**Published:** 2025-12-27

**Authors:** Xiaoyan Li, Bin Lv, Shumei Wang

**Affiliations:** 1 College of Traditional Chinese Medicine, Chongqing Medical University, Chongqing, 400016 China; 2 Department of Encephalopathy, Chongqing Qijiang Hospital of Traditional Chinese Medicine, Chongqing, 401420 China; 3 Orthopedics Department, Chongqing Qijiang Hospital of Traditional Chinese Medicine, Chongqing, 401420 China; 4 College of Chinese and Modern Medicine, Chongqing University of Chinese Medicine, Chongqing, 402760 China

**Keywords:** Chronic obstructive pulmonary disease, Bufei decoction, Schizandrin b, TLR4, Inflammatory response

## Abstract

**Objective:**

Evaluate Bufei Decoction effects on chronic obstructive pulmonary disease (COPD) and elucidate Schisandrin B (Sch B) mechanism via TLR4 pathway.

**Methods:**

COPD was induced in rats using LPS/cigarette smoke. Effects of low, medium, high-dose Bufei Decoction and doxofylline were assessed on lung pathology, function, blood gases, and inflammation. Sch B role was investigated via network pharmacology (identifying active components/targets), molecular docking (schisandrin B-TLR4 binding), and in vivo validation.

**Results:**

Bufei Decoction dose-dependently ameliorated COPD-induced lung injury, improved pulmonary function/blood gases, and reduced inflammation (high-dose most effective). Network pharmacology identified 241 Bufei Decoction components (including Sch B) targeting core molecules (TLR4, JAK1, STAT3). Molecular docking confirmed strong Sch B-TLR4 binding. Functional analysis implicated immune/inflammatory pathways. In vivo experiments showed that Sch B dose-dependently alleviated lung injury, improved pulmonary function, reduced the levels of inflammatory markers, and inhibited the M1/M2 macrophage ratio as well as the expression of proteins related to the NF-κB/JAK-STAT signaling pathway. Crucially, TLR4 knockdown reversed Sch B’s protective effects, worsening injury and inflammation.

**Conclusions:**

Bufei Decoction treats COPD through multi-component synergy. Sch B is a key active component, exerting therapeutic effects by targeting TLR4 to regulate macrophage polarization and inhibit the NF-κB/JAK-STAT signaling pathway, thereby improving lung function and reducing inflammation.

**Supplementary Information:**

The online version contains supplementary material available at 10.1186/s41065-025-00629-8.

## Introduction

Chronic Obstructive Pulmonary Disease (COPD) is a chronic heterogeneous airway disease characterized by persistent and progressive airflow limitation [1]. As predicted by the World Health Organization (WHO), this disease is expected to become the third leading cause of death worldwide by 2030 [2]. Although existing therapeutic agents such as bronchodilators and glucocorticoids can relieve clinical symptoms, COPD remains an incurable condition at present. Therefore, the development of safe and effective novel treatment options that can maintain long-term clinical stability, reduce the frequency of acute exacerbations, and shorten the course of acute exacerbation episodes is of critical importance for improving patients’ quality of life and clinical prognosis [3].

The primary etiological factors of COPD include air pollution, cold temperature exposure, and tobacco smoke [4]. Although the specific mechanisms of COPD remain under investigation, it is widely accepted that the release of inflammatory mediators and immune dysregulation form the basis of its pathogenesis. Sustained chronic inflammation triggers airway remodeling and narrowing by infiltrating and destroying normal tissues of the airways, leading to small airway obstruction and increased mucus secretion from lung tissue [5]. Macrophages, neutrophils, T lymphocytes, and epithelial cells serve as the main participants in COPD inflammatory responses [6]. Macrophages can be differentiated into specific subtypes upon stimulation by signals such as interferon-γ (IFN-γ) and lipopolysaccharide (LPS). Abnormal macrophage polarization has been confirmed as a key link driving COPD inflammation progression and lung tissue damage [7]. Activation of the nuclear factor-kappa B (NF-κB) signaling pathway via oxidative stress leads to the release of substantial reactive oxygen species (ROS) and induces the expression of proinflammatory cytokines [Interleukin-1 beta (IL-1β), interleukin-6 (IL-6), Tumor Necrosis Factor-alpha (TNF-α)] [8] as well as anti-inflammatory cytokines [interleukin-4 (IL-4), interleukin-10 (IL-10), interleukin-13 (IL-13)] [9, 10]. As a key transcription factor, NF-κB activation not only exacerbates tissue damage and recruits inflammatory cells but also propels the chronic inflammatory process by amplifying proinflammatory signaling cascades [11]. Emerging evidence indicates that the NF-κB pathway synergistically regulates the pulmonary inflammatory microenvironment through crosstalk with multiple signaling pathways, including mitogen-activated protein kinase (MAPK), phosphatidylinositol 3-kinase-Akt (PI3K-Akt), and Janus kinase-signal transducer and activator of transcription (JAK-STAT) [12, 13]. Additionally, in COPD, the JAK-STAT signaling pathway directly induces the expression of multiple proinflammatory genes, further exacerbating airway inflammatory responses [14–16].

In traditional Chinese medicine (TCM), COPD is categorized under " lung distension” and “dyspnea syndrome”. It is believed that its primary pathogenesis involves “phlegm, deficiency, and blood stasis”, with the pathological locations involving the Lung, Spleen, and Kidney. Bufei Decoction, a classic TCM formula, exerts effects in tonifying the Lung and Kidney, resolving phlegm, and relieving cough [17]. It has been demonstrated to modulate inflammatory responses, inhibit the release of proinflammatory cytokines, and delay the progression of COPD [18]. However, most existing studies have been limited to observing the holistic therapeutic effects of Bufei Decoction as a compound prescription. Systematic verification of the “compound-monomer-target-pathway” association has been lacking. The core material basis for its anti-COPD effects remains unclear.

Schisandrin B (Sch B), as the core active component of Bufei Decoction, has been mainly focused on mechanism studies such as anti-inflammation, antioxidation, and anti-pulmonary fibrosis. Particularly, favorable protective effects have been demonstrated in models like acute lung injury, asthma, and pulmonary fibrosis [19]. However, its anti-inflammatory and antioxidative mechanisms in the context of COPD have not been explored. Taking Bufei Decoction and its core active component Sch B as the entry points, this study established a rat COPD model induced by LPS combined with cigarette smoke exposure. The therapeutic effects and potential synergistic mechanisms of both on COPD were systematically investigated. For the first time, the core mechanism of Sch B in improving COPD was clarified, targeting TLR4 to regulate the NF-κB/JAK-STAT signaling pathway and balance macrophage polarization. This study provides new theoretical basis and potential drug targets for the TCM treatment of COPD.

## Materials and methods

### Animal husbandry and grouping

The animal experimental protocols involved in the study were reviewed and approved by the Institutional Animal Care and Use Committee of Chongqing Medical University (IACUC-CQMU), with the approval number: IACUC-CQMU-2024-0511. Twelve-month-old specific pathogen-free healthy Sprague-Dawley rats (body weight: 200 ± 50 g) were purchased from Chongqing Ensiweier Biotechnology Co., Ltd. The rats were acclimatized for 7 days in a controlled environment with a room temperature of 23 ± 2 °C, humidity of 60 ± 5%, and ad libitum access to food and water in a clean, ventilated facility. Daily observations were conducted to monitor coat condition, appetite, and behavioral status. Prior to the experiment, body weights were measured using an electronic scale to confirm no statistically significant differences in baseline weight among groups.

### COPD model construction

On day 1 and day 14, rats were anesthetized with 4% pentobarbital sodium (40 mg/kg, P3761, BSZH, Beijing, China). A 1-mL dead-space-free syringe was used to puncture the trachea, and 200 µL of LPS (1 g/L, L861706, Macklin, Shanghai, China) was instilled intratracheally. Immediately after injection, the animals were held upright and gently shaken for approximately 2 min to ensure uniform solution distribution in both lungs. The neck incision was then sutured, and the local area was disinfected, with strict aseptic techniques maintained throughout the procedure. From day 2 to day 30, rats were placed in a 50 cm × 40 cm × 40 cm glass smoking exposure chamber. A mixture of appropriate sawdust and tobacco shreds from 20 cigarettes was ignited for smoking, with 30 min of exposure in both the morning and afternoon daily, and 2 days of rest per week. Control group rats received intratracheal instillation of an equal volume of 0.9% saline at corresponding time points.

### Drug preparation

Crude drugs of the Bufei Decoction prescription were prepared with impurities removed and crushed into coarse powder. Ten times the volume of distilled water was added for soaking for 30 min, followed by continuous decoction in a stainless-steel decoction pot for 60 min. After decoction, the supernatant was collected by filtration through gauze. Eight times the volume of distilled water was added to the dregs for a second decoction of 40 min, and the supernatant was collected again by filtration. The two supernatants were combined and concentrated to a crude drug concentration of 1800 mg/mL using a rotary evaporator (RE-52AA, YaRong, Shanghai, China) under vacuum conditions (55 °C, -0.08 MPa). The concentrated medicinal solution was sterilized by filtration through a 0.22 μm microporous membrane, aliquoted into sterile reagent bottles, and stored at 4 °C. It was brought to room temperature before use. The high (18000 mg/kg), medium (9000 mg/kg), and low (4500 mg/kg) doses of Bufei Decoction were set based on a study by Zhang et al. [18], which confirmed that 4310 mg/kg Bufei Decoction could significantly improve pulmonary function, reduce lung tissue inflammation and collagen deposition in COPD rats induced by lipopolysaccharide combined with cigarette smoke. The low dose (4500 mg/kg) in this study was close to this effective dose. Referring to the Table of Drug Dosage Conversion Between Laboratory Animals and Humans, the clinical equivalent dose for rats was calculated using the body surface area conversion formula (rat dose = human dose × rat body surface area coefficient 0.012 / human body surface area coefficient 1.62), based on the conventional clinical dosage of Bufei Decoction for adults (equivalent to 90000 mg/day of crude drug for a 60-kg adult). The calculated clinical equivalent dose range for rats was 8100–10,800 mg/kg, and the medium dose (9000 mg/kg) in this study was highly consistent with this range. Doxofylline Tablets (National Drug Approval Number H20000076, Tianheng Pharmaceutical Co., Ltd., Zhejiang, China) were ground into fine powder. Referring to the above conversion table and formula, the equivalent dose for rats was calculated as 8.6 mg/kg based on the clinical human dosage of doxofylline (800 mg/day for a 60-kg adult). Combined with a gavage volume of 10 mL/kg, the doxofylline fine powder was dissolved in sterile normal saline to prepare a 0.86 mg/mL solution. The solution was filtered through a 0.22 μm microporous membrane, aliquoted into sterile centrifuge tubes, and freshly prepared before use.

### Experimental groups and drug administration

After model establishment and before drug administration, two model rats were randomly selected for euthanasia. Lung tissues were harvested for hematoxylin and eosin (HE) staining to observe gross morphological changes, including lung swelling, pallor, increased volume, interstitial inflammatory cell infiltration, alveolar enlargement, structural disorder, and thinning/rupture of alveolar walls. Upon successful modeling, thirty COPD model rats were divided into five groups (*n* = 6 per group, equal male-female ratio) using a random number table method for 28 days of treatment. The specific grouping was as follows: Model group: Gastric gavage with normal saline; L-Bufei Decoction group: Gastric gavage with Bufei Decoction at 4500 mg/kg; M-Bufei Decoction group: Gastric gavage with Bufei Decoction at 9000 mg/kg; H-Bufei Decoction group: Gastric gavage with Bufei Decoction at 18,000 mg/kg; Doxofylline group: Gastric gavage with doxofylline sustained-release tablets (positive control). Additionally, six healthy rats served as the control group, receiving normal saline via gastric gavage.

### General condition observation of rats

After the administration intervention, the nutritional status of rats was observed, including the luster of their fur and the presence or absence of hair loss. Respiratory rate was monitored, along with the occurrence of coughing, shortness of breath, wheezing, and other abnormal respiratory conditions. Food and water intake, as well as feces and urine output, were carefully recorded. The presence of abnormal secretions in the nasal cavity was examined. Additionally, the mental state, activity level, and stress responses to external stimuli of the rats were observed. All the above-mentioned general conditions were separately recorded, and the body weight and body temperature of each group of rats were measured.

### HE staining

The lung tissues of rats were processed into paraffin sections. The sections were first dewaxed with xylene and then rehydrated through a series of gradient ethanol solutions. Subsequently, they were stained with hematoxylin staining solution (G1004, Servicebio, Wuhan, China) for 5 min, followed by decolorization with a differentiation solution. After that, the sections were stained with eosin staining solution (G1002, Servicebio, Wuhan, China) for 2 min. Then, the sections underwent dehydration using gradient ethanol solutions and transparency treatment with xylene. Finally, the sections were mounted with neutral resin (10004160, Sinopharm, Beijing, China). Images of the stained sections were acquired using a 3DHistech Pannoramic Scanner (Pannoramic MIDI II, 3DHistech, Hungary), an automatic digital slide scanner. Based on the above-mentioned HE-stained section images, the degree of lung tissue injury was evaluated according to the following criteria.The evaluation items include six items [20]: edema, atelectasis, necrosis, alveolar and interstitial inflammation, hemorrhage, and hyaline membrane formation. Each item was scored according to the extent of injury: 0 point for no injury, 1 point for 25% area injured, 2 points for 50% area injured, 3 points for 75% area injured, and 4 points for 100% area injured. During scoring, 10 fields of view at 200× magnification were randomly selected from each section, and the degree of injury for each of the above items was scored separately.

### Pulmonary function test

An automated pulmonary function test and data analysis of rats were performed using the DSI Buxco^®^ Pulmonary Function Testing System (PFT, DSI Buxco^®^, Beijing, China). Before the test, the calibration procedures for functional residual capacity flow, high-flow, and pulmonary pressure curves were strictly carried out by the system operation manual to ensure that the equipment parameters were in a standard state. The rats were maintained at a moderate anesthesia level. After establishing an airway pathway through tracheal intubation, the rats were gently placed into the plethysmograph chamber and allowed to rest for 2 min to adapt to the chamber environment. Once the respiratory rate of the rats stabilized, the tracheal catheter was precisely connected to the built-in breathing valve of the chamber. The chamber lid was closed to form a closed space, and then the system was initiated to automatically collect all parameters.

### Arterial blood gas analysis

The arterial blood gas samples of rats were synchronized with a veterinary blood gas analyzer (Derry D15 VET, Shenzhen, China) from Shenzhen Cutting-edge Technology Co. Before the operation, the rats were confirmed to be under moderate anesthesia, fixed in the supine position on the operating table, skin preparation of the thorax, abdomen and right lower limb, disinfection with iodophor were done sequentially. The femoral artery of the right lower limb was surgically dissected and exposed. A disposable sterile 24G indwelling cannula needle was used for femoral artery puncture. After a successful puncture, the needle body was properly fixed with medical adhesive tape. The needle was then connected to a pressure transducer and linked to a multi-channel physiological signal acquisition system. The zero point was calibrated in real-time, and the arterial blood pressure waveform was monitored.

### Bronchoalveolar Lavage Fluid (BALF) collection

Rats were anesthetized via intraperitoneal injection and then secured in a supine position on a surgical board. The femoral artery of the right hind limb was isolated and used for exsanguination. The thoracic cavity was then carefully opened layer by layer to fully expose the trachea. A small transverse incision (approximately 1 mm) was made in the anterior wall of the lower trachea. This incision was then extended longitudinally in a cephalad direction, creating a T-shaped structure. A blunt-ended stainless steel lavage needle with an outer diameter of 1 mm was inserted into the trachea through this opening. Using a 2 mL sterile syringe, 1 mL of pre-warmed (37 °C) 0.9% sodium chloride solution was slowly injected into the lungs via the lavage needle. Gradual expansion and whitening of the lung tissue was observed as the fluid was instilled. After allowing the fluid to dwell in the lungs for 5 s, the BALF was gently aspirated at a rate of 0.3 mL/s. This lavage-aspiration cycle was repeated three times, for a total lavage volume of 3 mL. All recovered BALF was collected into a 10 mL sterile centrifuge tube and centrifuged at 4000 × g for 5 min at 4 °C. The supernatant was then transferred to pre-chilled 1.5 mL Eppendorf (EP) tubes, aliquoted into 0.5 mL volumes per tube, and immediately stored at -80 °C in an ultra-low temperature freezer until further analysis.

### Enzyme-Linked Immunosorbent Assay (ELISA)

The levels of rat interleukin-4 (IL-4) (JL20894-96T, Jonlnbio, Shanghai, China), rat interleukin-6 (IL-6) (PI328, Beyotime, Shanghai, China), rat interleukin-8 (IL-8) (TW1571, TONGWEI, Shanghai, China), and tumor necrosis factor-alpha (TNF-α) (PT516, Beyotime, Shanghai, China) were quantified in the BALF supernatant using commercially available ELISA kits. The assays were performed according to the manufacturer’s instructions for each respective kit.

### Network Pharmacology analysis

The structural file of Bufei Decoction was obtained from the Traditional Chinese Medicine Systems Pharmacology Database and Analysis Platform (TCMSP) (http://lsp.nwu.edu.cn/tcmsp.php). The file was then imported into the SwissTargetPrediction database, and the drug targets were predicted with a probability threshold > 0. Additionally, the targets of Bufei Decoction were screened using the BatmAn-TCM 2.0 database with a score cutoff > 0.84. The targets collected from the above databases were combined, and duplicate items were removed to obtain the predicted targets of Bufei Decoction. Subsequently, the Cytoscape 3.7.2 software was utilized to construct a network diagram of drugs, active ingredients, and targets.​Using “Chronic Obstructive Pulmonary Disease” as the keyword, potential targets of COPD were predicted in the GeneCards (https://www.genecards.org/), DisGeNET (https://www.disgenet.org/), DrugBank (https://www.drugbank.ca/), Online Mendelian Inheritance in Man (OMIM, https://www.omim.org/), and UniProt (https://www.uniprot.org) databases. Targets were screened with a relevance score > 10. Then, the Jvenn online website (https://www.bioinformatics.com.cn) was employed to perform an intersection analysis on the predicted targets of Bufei Decoction and COPD. The intersection results were further intersected with the drug target dataset to screen out the drug-disease targets, and a Venn diagram was drawn.​.

### Construction of the Protein-Protein interaction (PPI) network

The obtained intersection targets of Bufei Decoction and COPD were input into the Search Tool for the Retrieval of Interacting Genes/Proteins (STRING) database (http://string-db.org/cgi/input.pl). The tab-separated values (tsv) file was downloaded and then imported into the Cytoscape 3.7.2 software for visualization processing. The CytoNCA plugin was utilized to conduct an analysis of the network topological structure, and the CytoHubba extension program was employed to calculate the node scores.

### Molecular docking

The three-dimensional structures of the ligands were downloaded from the PubChem database (https://pubchem.ncbi.nlm.nih.gov) and optimized using the SYBYL-X 2.0 software (https://www.rcsb.org). The three-dimensional structures of the receptors were sourced from the Research Collaboratory for Structural Bioinformatics (RCSB) Protein Data Bank (https://www.rcsb.org). The AutoDock Vina 1.1.2 software (Download vina_split (AutoDock Vina 1.1.2–64-bit)) was employed to evaluate the binding affinity between the compounds and the target proteins. Subsequently, the docking results were visualized using the Discovery Studio software.

### Functional enrichment analysis

The R packages such as “ggplot2” in R language (Version 4.0.1, www.r-project.org) were used to conduct Gene Ontology (GO) and Kyoto Encyclopedia of Genes and Genomes (KEGG) enrichment analyses on the intersection targets. A significance level of *p* < 0.05 was considered to indicate statistical differences.

### Intervention of COPD model with monomer Sch B

Sch B (B21327, Yuanye, Shanghai, China) was dissolved in 0.9% sterile normal saline as the solvent to prepare stock solutions with concentrations of 25 mg/mL (low dose), 40 mg/mL (medium dose), and 50 mg/mL (high dose), respectively. All stock solutions were sterilized by filtration through a 0.22 μm polyethersulfone (PES) membrane and stored at 4 °C in a light-protected environment. The intervention doses of Sch B (25 mg/kg/d, 40 mg/kg/d, 50 mg/kg/d) were set based on two core preclinical studies: Referring to the report by Li et al. [21], which confirmed that Sch B in the dose range of 20–60 mg/kg could activate the Nrf2/HO-1 pathway to alleviate pulmonary oxidative stress and inflammatory damage in a rat model of acute lung injury. The high dose (50 mg/kg) and medium dose (40 mg/kg) in this study were both within this effective range. Combined with the research conclusions by Cai et al. [22], who found that 25 mg/kg Sch B could reduce the lung wet/dry weight ratio and myeloperoxidase (MPO) activity in a mouse model of lung injury, the low dose was set at 25 mg/kg. The medium dose (40 mg/kg) was used as a transitional gradient for the dose-effect relationship to cover the complete effect range and avoid potential risks of high doses. After the successful verification of the COPD models, gavage administration was carried out according to the grouping, with 9 rats in each group. The administration was conducted once a day for 30 consecutive days. The specific grouping is as follows: Model group: Given normal saline by gavage; L-Sch B group: The COPD models were given Sch B by gavage at a dose of 25 mg/kg/d; M-Sch B group: The COPD models were given Sch B by gavage at a dose of 40 mg/kg/d; H-Sch B group: The COPD models were given Sch B by gavage at a dose of 50 mg/kg/d; Doxofylline group: The COPD models were given doxofylline tablets by gavage. In addition, 9 healthy rats were selected as the control group and were given normal saline by gavage.

### In vivo verification of the target gene TLR4

The Chongqing Boaimedicine Biotechnology Co., Ltd. designed the specific short-hairpin RNA (shRNA) sequence for TLR4 and the negative control sequence (shRNA - NC). These sequences were cloned into an adeno-associated virus (AAV) vector. After packaging in 293T cells, the virus titer was determined to be 1 × 10¹² Genome Copy per milliliter (GC/mL). Before use, the virus was diluted to a working concentration of 1 × 10¹¹ GC/mL with sterile phosphate - buffered saline (PBS). Then, 3 µL of the virus solution was slowly dropped into the bilateral nostrils of the successfully established COPD rat models. Meanwhile, the rats in the model group were treated with Sch B by gavage at a dose of 50 mg/kg/d.The specific grouping was as follows: Control group: Healthy rats were given normal saline by gavage and normal saline drops in the nostrils. Model group: COPD model rats were given normal saline by gavage and normal saline drops in the nostrils. Sch B group: COPD model rats were given Sch B by gavage (50 mg/kg/d) and normal saline drops in the nostrils. Sch B + shRNA-TLR4-NC group: COPD model rats were given Sch B by gavage (50 mg/kg/d) and drops of the TLR4 negative-control virus in the nostrils. Sch B + shRNA-TLR4 group: COPD model rats were given Sch B by gavage and drops of the TLR4-interfering virus in the nostrils. Doxofylline group: COPD model rats were given doxofylline tablets by gavage and normal saline drops in the nostrils.

### Real-time quantitative polymerase chain reaction (RT-qPCR)

The total RNA was extracted from the samples using the Trizol method, and the quality of the RNA was assessed using an ultraviolet - visible spectrophotometer (NanoDrop One/One C, Thermo, Massachusetts, USA). The RNA was reverse - transcribed into complementary DNA (cDNA) using the Goldenstar™ RT6 cDNA Synthesis Kit Ver.2 (TSK302M, Tsingke, Beijing, China). The quantitative polymerase chain reaction (qPCR) experiments were performed using the 2 × T5 Fast qPCR Mix (SYBR Green I) (TSE202, Tsingke, Beijing, China). Glyceraldehyde − 3 - phosphate dehydrogenase (GAPDH) was used as the internal reference gene. Primer sequences in Table 1.

**Table 1:** Primer sequences used in qPCR

Primer name	Sequences
TLR4-F	GCTCTTGGTGGAAGTTGAACGAATG
TLR4-R	CAAGCACACTGAGGACCGACAC
iNOS-F	TGGAGCGAGTTGTGGATTGT
iNOS-R	GTAGTGATGTCCAGGAAGTAGGT
CD86-F	AAGACATGTGTAACCTGCACCA
CD86-R	ACTTTTTCCGGTCCTGCCAA
Arg1-F	GAGAAGGTCTCTACATCACAGAAG
Arg1-R	TTCACAGTACGAGTCACCTCC
CD206-F	TGGACAGACGGACGAGGAGTTC
CD206-R	GCCACCAATCACAACAACACAGTC
P65-F	CACCAAAGACCCACCTCACCG
P65-R	CTTGCTCCAGGTCTCGCTTC
GAPDH-F	CAATCCTGGGCGGTACAACT
GAPDH-R	TACGGCCAAATCCGTTCACA

Inducible nitric oxide synthas, iNOS; Cluster of differentiation 86, CD86; Arginase 1, Arg1; Cluster of differentiation 206, CD206

### Western Blotting (WB)

To extract proteins, RIPA lysis buffer containing phenylmethylsulfonyl fluoride (PMSF) (ST507, Beyotime, Shanghai, China) and protease inhibitor cocktail (P1045, Beyotime, Shanghai, China) was added to the samples. The protein concentration was determined using the BCA protein assay kit (P0012, Beyotime, Shanghai, China). Total protein samples were mixed with 5× SDS loading buffer (8015011, DAKEWE, Shenzhen, China), denatured by heating, and subjected to electrophoresis. Proteins were separated on a gel, initially at 80 V through the stacking gel, followed by 120 V. Separated proteins were then transferred to a membrane at a constant current of 250 mA. Membranes were blocked with 5% non-fat milk (P0216, Beyotime, Shanghai, China) and incubated overnight at 4 °C with primary antibodies diluted to the appropriate concentration. After washing, membranes were incubated with a secondary antibody (AS014, abclonal, Wuhan, China) for 1 h at room temperature. Following further washes, an enhanced chemiluminescence (ECL) substrate (34580, Thermo, Waltham, Massachusetts, USA) was applied for 1 min, and the membranes were imaged using a Universal Hood II imaging system (Bio-Rad, Hercules, California, USA). The primary antibodies used were: iNOS (A14031, ABclonal, Wuhan, China), TLR4 (ABclonal, Wuhan, China), STAT1 A19563, ABclonal, Wuhan, China), STAT3 (A22434, ABclonal, Wuhan, China), Arg1 (A1847, ABclonal, Wuhan, China), and P65 (A19653, ABclonal, Wuhan, China). GAPDH (A19056, ABclonal, Wuhan, China) served as the loading control. Densitometric analysis of protein bands was performed using ImageJ software (version 1.8.0, https://imagej.en.softonic.com/download).

### Statistical analysis

The data obtained from the experiments were analyzed using GraphPad Prism 8.0.1 software (version 8.0.1, GraphPad Software, USA, https://www.graphpad-prism.cn), and the figures were formatted and created with Adobe Illustrator 2021 software (version 2021, Adobe, USA, https://www.adobe.com/cn/products/illustrator.html). Comparisons among groups were performed using one-way analysis of variance (ANOVA) and t-tests. All experimental procedures were repeated at least three times. Statistical significance was set at a criterion of *p* < 0.05.

## Results

### Validation of the COPD model

**Fig. 1:**
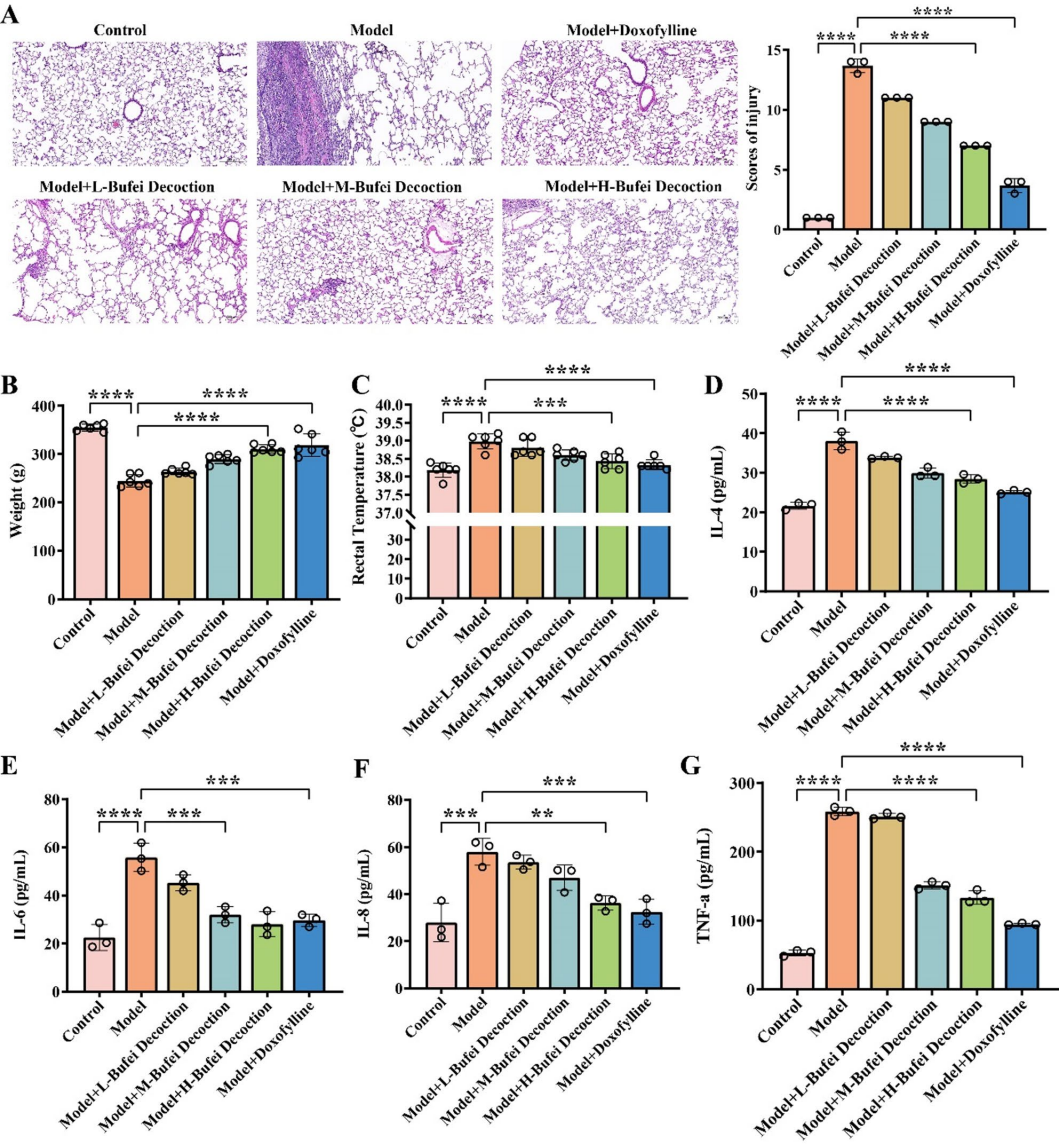
Validation of COPD rat models. **A** HE staining of rat lung tissues and scores of injury (Scale bar: 200 μm). **B** Body weight measurements of rats before and after model establishment and in each experimental group. **C** Rectal temperature measurements of rats before and after model establishment and in each experimental group. **D**-**G** Detection of IL − 4, IL − 6, IL − 8, and TNF–α cytokines in the bronchoalveolar lavage fluid of rats before and after model establishment and in each experimental group by ELISA. All data are presented as mean ± standard deviation (mean ± SD). *N* = 6; **, *p* < 0.01; ***, *p* < 0.001; ****, *p* < 0.0001

**Fig. 2:**
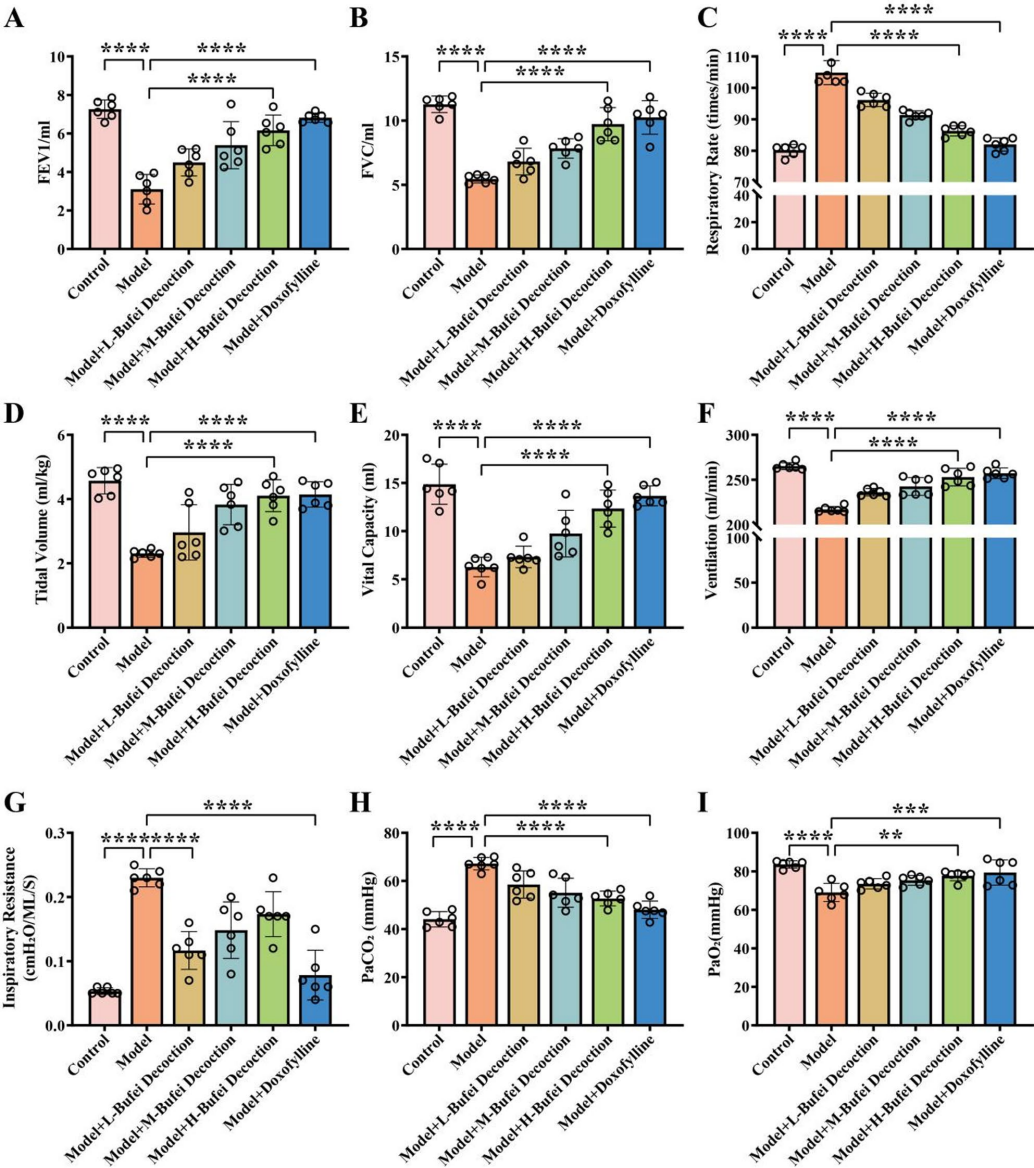
Pulmonary function and arterial blood gas tests in COPD rats. **A** - **G** Measurement of FEV1, FVC, respiratory rate, tidal volume, vital capacity, minute ventilation volume, and inspiratory resistance of rats before and after model establishment and in each experimental group. **H**-**I** Detection of partial pressure of PaCO_2_ and PaO_2_ of rats before and after model establishment and in each experimental group. All data are presented as mean ± SD. *N* = 6; **, *p* < 0.01; ****, *p* < 0.0001

To verify the successful establishment of the COPD model, this study comprehensively evaluated the pathological changes of lung tissues, body weight, body temperature, pulmonary function indices, arterial blood gas analysis results, and inflammatory factor levels in COPD model rats. The results of HE staining (Fig. 1A) showed that the lung tissues of rats in the model group exhibited significant pathological damage (*p* < 0.0001), including disordered, widened, and thickened alveolar walls, as well as massive infiltration of lymphocytes and inflammatory cells. This indicated that the COPD model induced obvious pulmonary inflammation and structural changes. Compared with the control group, the body weight of rats in the model group decreased (*p* < 0.0001) (Fig. 1B), the body temperature increased (*p* < 0.0001) (Fig. 1C), and the forced expiratory volume in one second (FEV1) (Fig. 2A), forced vital capacity (FVC) (Fig. 2B), tidal volume (Fig. 2D), vital capacity (Fig. 2E), and minute ventilation volume (Fig. 2F) decreased (*p* < 0.0001). In contrast, the respiratory rate (Fig. 2C) and inspiratory resistance (Fig. 2G) increased (*p* < 0.0001), the partial pressure of carbon dioxide in arterial blood (PaCO_2_) rose (*p* < 0.0001) (Fig. 2H), and the partial pressure of oxygen in arterial blood (PaO2) decreased (*p* < 0.0001) (Fig. 2I). The levels of IL-4 (*p* < 0.0001) (Fig. 1D), IL − 6 (*p* < 0.001) (Fig. 1E), IL-8 (*p* < 0.01) (Fig. 1F), and TNF-α (*p* < 0.0001) (Fig. 1G) in the bronchoalveolar lavage fluid all increased. Collectively, the above results indicate that the COPD rat model induced by the combination of LPS and cigarette smoke was successfully established, presenting typical pulmonary inflammation, airflow limitation, and pulmonary gas exchange dysfunction.

### Bufei Decoction improves pulmonary function and inflammatory responses in COPD rats

The therapeutic effects of Bufei Decoction on COPD rats were further evaluated. As shown in Fig. 1A, compared with the model group, pulmonary pathological damage was alleviated in rats treated with different doses of Bufei Decoction (*p* < 0.0001). Specifically, the degree of alveolar wall thickening decreased, and the infiltration of inflammatory cells was reduced. The high-dose group exhibited a more pronounced improvement (*p* < 0.0001), indicating that Bufei Decoction has a protective effect on the lung tissues of COPD rats in a dose-dependent manner. In addition, after Bufei Decoction intervention, the body weight of rats in each dose group increased in a dose - dependent manner (Fig. 1B), while the body temperature decreased dose - dependently (Fig. 1C). FEV1 (Fig. 2A), forced vital capacity (FVC) (Fig. 2B), tidal volume (Fig. 2D), vital capacity (Fig. 2E), and minute ventilation volume (Fig. 2F) increased with the increase in the dose of Bufei Decoction (*p* < 0.0001). Conversely, the respiratory rate (Fig. 2C) and inspiratory resistance (Fig. 2G) gradually decreased (*p* < 0.0001). Moreover, the partial pressure of carbon dioxide PaCO2 (Fig. 2H) decreased significantly, and the partial pressure of oxygen in PaO2 (Fig. 2I) increased significantly (*p* < 0.01) with the increasing dose of Bufei Decoction. These findings suggest that Bufei Decoction may improve respiratory function and pulmonary gas exchange by ameliorating COPD-related metabolic disorders and reducing inflammatory responses. The group treated with doxofylline tablets also showed alleviated lung tissue damage, increased body weight, and decreased body temperature. Meanwhile, FEV1 (Fig. 2A), FVC (Fig. 2B), tidal volume (Fig. 2D), vital capacity (Fig. 2E), and minute ventilation volume (Fig. 2F) increased significantly (*p* < 0.0001), while the respiratory rate (Fig. 2C) and inspiratory resistance (Fig. 2G) decreased (*p* < 0.0001), PaCO2 decreased (Fig. 2H) (*p* < 0.0001), and PaO2 (Fig. 2I) increased (*p* < 0.001). The therapeutic effects of doxofylline were more significant than those of the high - dose Bufei Decoction group. Regarding the levels of inflammatory factors, after Bufei Decoction intervention, the levels of IL − 4 (Fig. 1D), IL − 6 (Fig. 1E), IL − 8 (Fig. 1F), and TNF - α (Fig. 1G) in the bronchoalveolar lavage fluid of rats in each dose group decreased in a dose - dependent manner (*p* < 0.05), indicating that Bufei Decoction may mitigate pulmonary inflammation by inhibiting the release of these inflammatory factors. Compared with the Bufei Decoction intervention groups, the doxofylline - treated group also showed reduced levels of inflammatory factors, and the decreases in IL − 4, IL − 6, IL − 8, and TNF - α were more pronounced than those in the high-dose Bufei Decoction group (*p* < 0.0001;*p* < 0.001༛*p* < 0.001༛*p* < 0.0001).

### Network Pharmacology analysis of Bufei Decoction

**Fig. 3:**
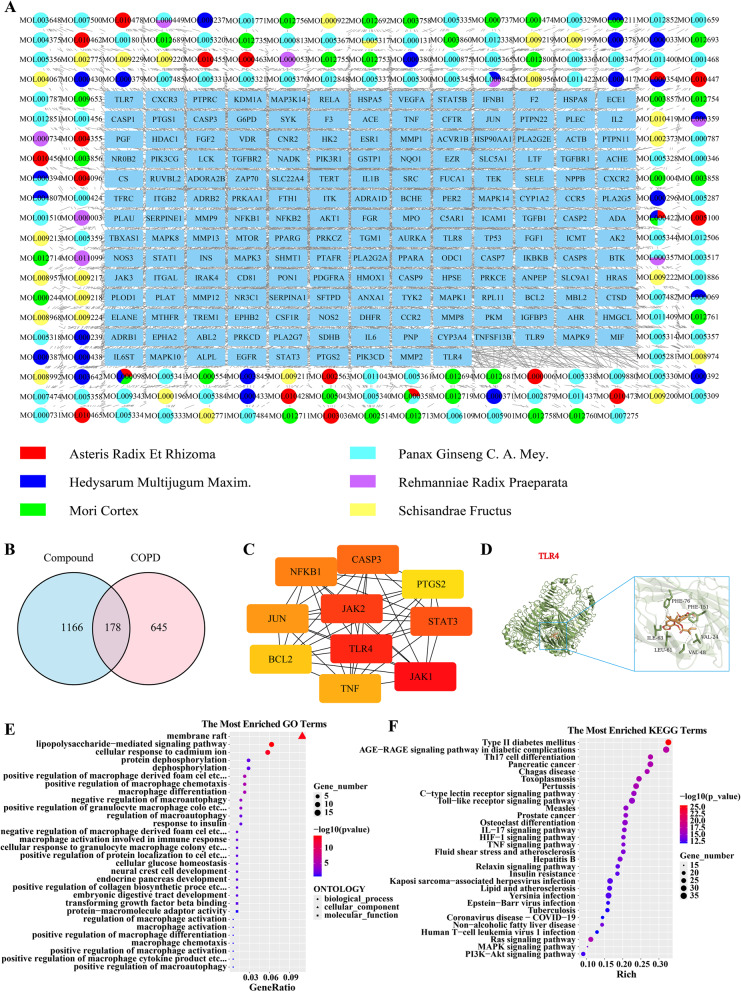
Network pharmacology analysis and molecular docking. **A** Drug-active ingredient-target network diagram. **B** Venn diagram of intersecting genes between drug-active ingredient targets and disease-related targets. **C** String analysis of protein interactions TOP10 network diagram. **D** Docking results of Lung tonic soup and TLR4. **E** Intersecting genes GO analysis bubble diagram. **F** Intersecting genes KEGG analysis Bubble diagram

Based on systematic screening using the TCMSP and HERB databases, a total of 241 major active components were identified in Bufei Decoction (Fig. 3A). The core components include *Panax ginseng* (92 components), *Morus alba root bark* (Folium Mori, 44 components), *Schisandra chinensis* (40 components), *Astragalus membranaceus* (34 components), *Aster tataricus* (20 components), and *Rehmannia glutinosa* (11 components). Notably, Sch B, a monomeric compound isolated from *Schisandra chinensis*, exhibits significant antifibrotic activity. After predicting the action targets of the active ingredients of lung tonic soup by Swiss Target Prediction platform, 178 intersecting gene targets were obtained by Venn analysis in combination with COPD-related targets screened by disease database (Fig. 3B). The STRING database was utilized to screen the top 10 core targets that were closely related to COPD treatment, which were Toll-like receptor 4 (TLR4), Janus kinase 1 (JAK1), Janus kinase 2 (JAK2), signal transducer and activator of transcription 3 (STAT3), caspase 3 (CASP3), nuclear factor kappa B subunit 1 (NFKB1), Jun proto-oncogene (JUN), tumor necrosis factor (TNF), B-cell lymphoma 2 (BCL2), and prostaglandin G/H synthase 2 (PTGS2). Among these, TLR4, a key regulator of the innate immune response, was preliminarily determined as the core target through which Bufei Decoction intervenes in COPD. Molecular docking results indicate that the interaction between TLR4 and Schizandrin B is mediated by specific amino acid residues, including VAL-24, VAL-48, LEU-61, ILE-63, PHE-76, and PHE-151. These residues are concentrated in the extracellular domain (ECD) of TLR4, specifically within the functionally critical region of its leucine-rich repeat (LRR) domain. From the perspective of amino acid properties, valine (VAL), leucine (LEU), and isoleucine (ILE) are hydrophobic amino acids. Phenylalanine (PHE) is both hydrophobic and aromatic. They form stable non-covalent interactions with the aromatic and alicyclic moieties of Schizandrin B via hydrophobic interactions and π-π stacking. In addition, the binding energy between them is − 7.7 kJ/mol. This energy level indicates that TLR4 can form a thermodynamically stable docking conformation with Schizandrin B (Fig. 3D).

### GO and KEGG enrichment analysis

GO functional enrichment analysis revealed that (Fig. 3E) in the category of Biological Processes (BP), genes were significantly enriched in immune-inflammatory regulatory pathways, including lipopolysaccharide-mediated signaling pathways, regulation of macrophage activation, differentiation, chemotaxis, and autophagy, as well as cellular responses to cadmium ions, protein dephosphorylation, and cellular glucose homeostasis. For Cellular Components (CC), enrichment was primarily observed in “membrane raft.” In Molecular Functions (MF), the enrichment focused on transforming growth factor beta binding and protein–macromolecule adaptor activity. The KEGG pathway enrichment analysis of the intersecting genes between Bufei Decoction and COPD shows that (Fig. 3F) in terms of immune and inflammatory regulation, it is significantly enriched in pathways such as the Toll-like receptor signaling pathway, Th17 cell differentiation, and IL-17 signaling pathway. This indicates that Bufei Decoction can intervene in the chronic inflammation of COPD by regulating the differentiation of immune cells and the inflammatory signaling network. In terms of the association between metabolism and comorbidities, it is enriched in pathways such as the AGE-RAGE signaling pathway in diabetic complications, Type II diabetes mellitus, and Non-alcoholic fatty liver disease. This suggests that its intervention in COPD may be related to improving metabolic disorders and regulating the microenvironment of comorbidities. In addition, in terms of the association between metabolism and comorbidities, pathways including Epstein-Barr virus infection, Tuberculosis, Pancreatic cancer, etc., are also involved. This reflects that Bufei Decoction may exert comprehensive effects through multi-system regulation such as anti-infection and anti-virus.

### Sch B alleviates inflammation in COPD rats

**Fig. 4:**
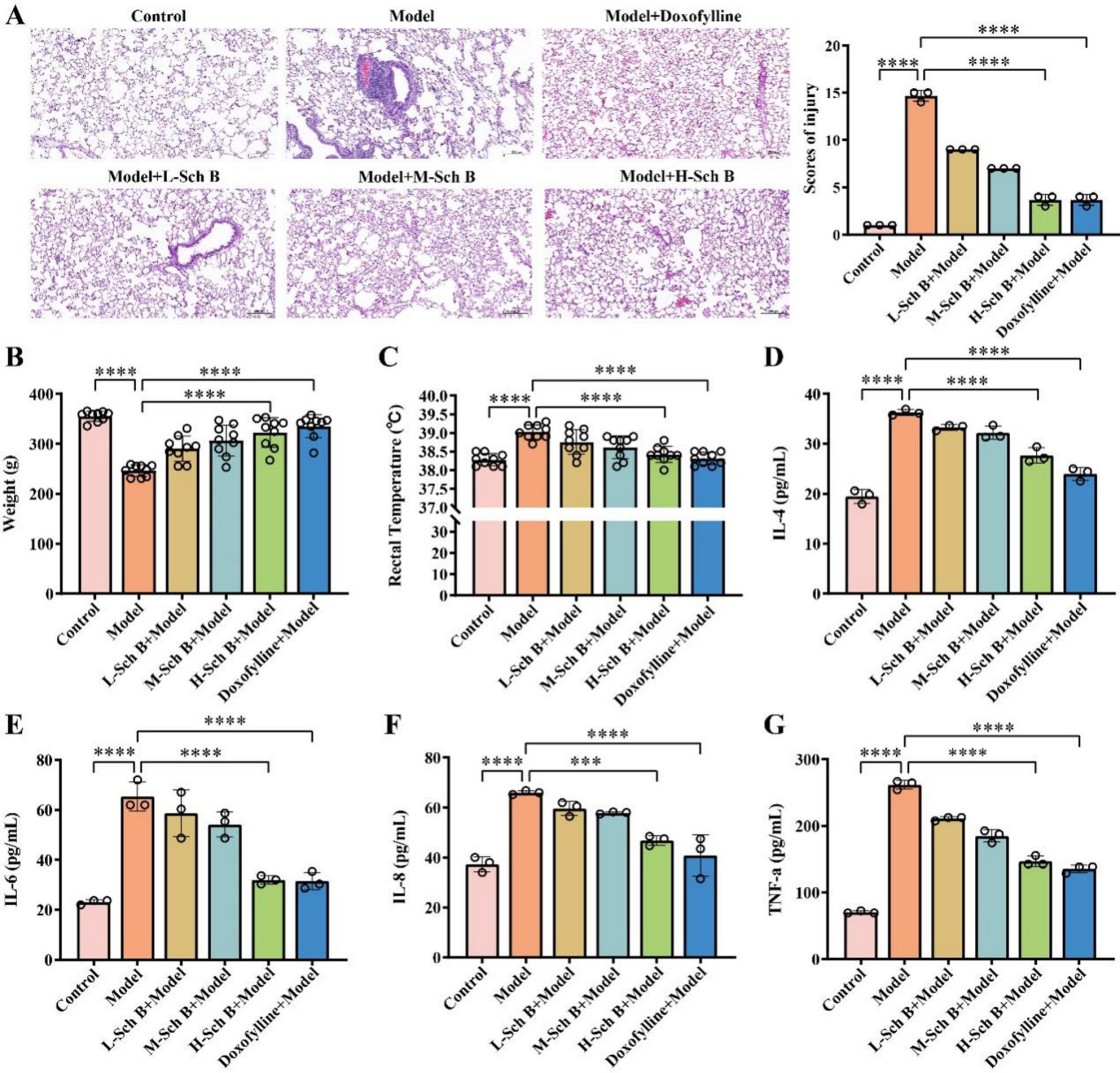
Sch B Alleviates Inflammation in COPD Rats. **A** HE staining of the lung tissues of rats and scores of injury (Scale bar: 200 μm). **B** Changes in the body weight of rats. **C** Detection of the rectal body temperature of rats. *N* = 9. **D**-**G** Detection of IL-4, IL-6, IL-8, and TNF-α in the bronchoalveolar lavage fluid of rats by ELISA. All data are presented as mean ± SD. *N* = 3; ***, *p* < 0.001; ****, *p* < 0.0001

To further validate the efficacy of Sch B, rats in the model group were administered different doses of Sch B via gavage in this study. The results of HE staining (Fig. 4A) showed that the alveolar structure in the control group was intact. In contrast, the model group exhibited severe disorder of the alveolar wall, which was widened and thickened, along with many infiltrating inflammatory cells. As the dose of Sch B increased, the morphological structure of the alveolar cavity in the low-dose group showed disorder, with a slight thickening of the alveolar wall and a relatively large number of infiltrating inflammatory cells. In the medium- and high-dose groups, the degree of morphological disorder of the alveolar cavity gradually decreased, and the thickening of the alveolar wall and the infiltration of inflammatory cells were significantly improved (*p* < 0.0001). This indicates that Sch B can reduce the pathological damage of lung tissues in COPD rats in a dose-dependent manner. In addition, as the dose of Sch B increased, the body weight of the rats in the model group gradually increased, and the body temperature decreased in a dose-dependent manner (*p* < 0.0001) (Fig. 4B-C). Moreover, compared with the model group, the levels of IL-4, IL-6, IL-8, and TNF-αin the bronchoalveolar lavage fluid of the rats in the groups intervened with different doses of Sch B significantly decreased in a dose-dependent manner(*p* < 0.0001) (Fig. 4D-G). Among them, as key pro-inflammatory cytokines, the levels of IL-6 and TNF-α decreased significantly, indicating that Sch B can effectively inhibit the core links in the inflammatory cascade reaction, which is highly consistent with the pathological changes in the lung tissues.

### Sch B ameliorates respiratory dysfunction and regulates macrophage polarization in COPD rats

**Fig. 5:**
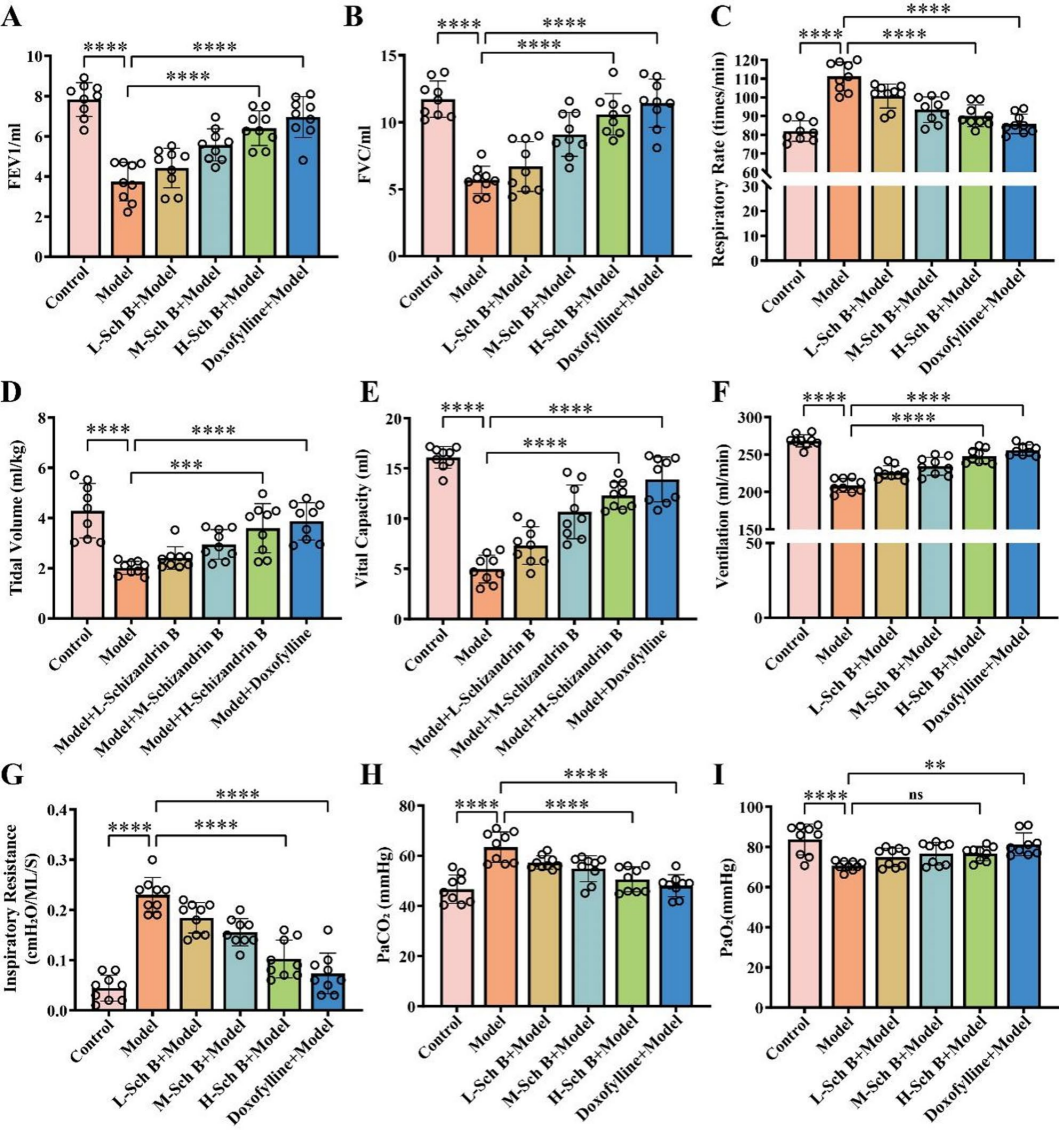
Detection of Respiratory Function in COPD Rats. **A**-**G** Measurement of FEV1, FVC, respiratory rate, tidal volume, vital capacity, minute ventilation volume, and inspiratory resistance in rats. **H**-**I** Detection of PaCO_2_ and PaO_2_ in rats. All data are presented as mean ± SD. *N* = 9; “*ns*” indicates no significance; **, *p* < 0.01; ***, *p* < 0.001; ****, *p* < 0.0001

**Fig. 6:**
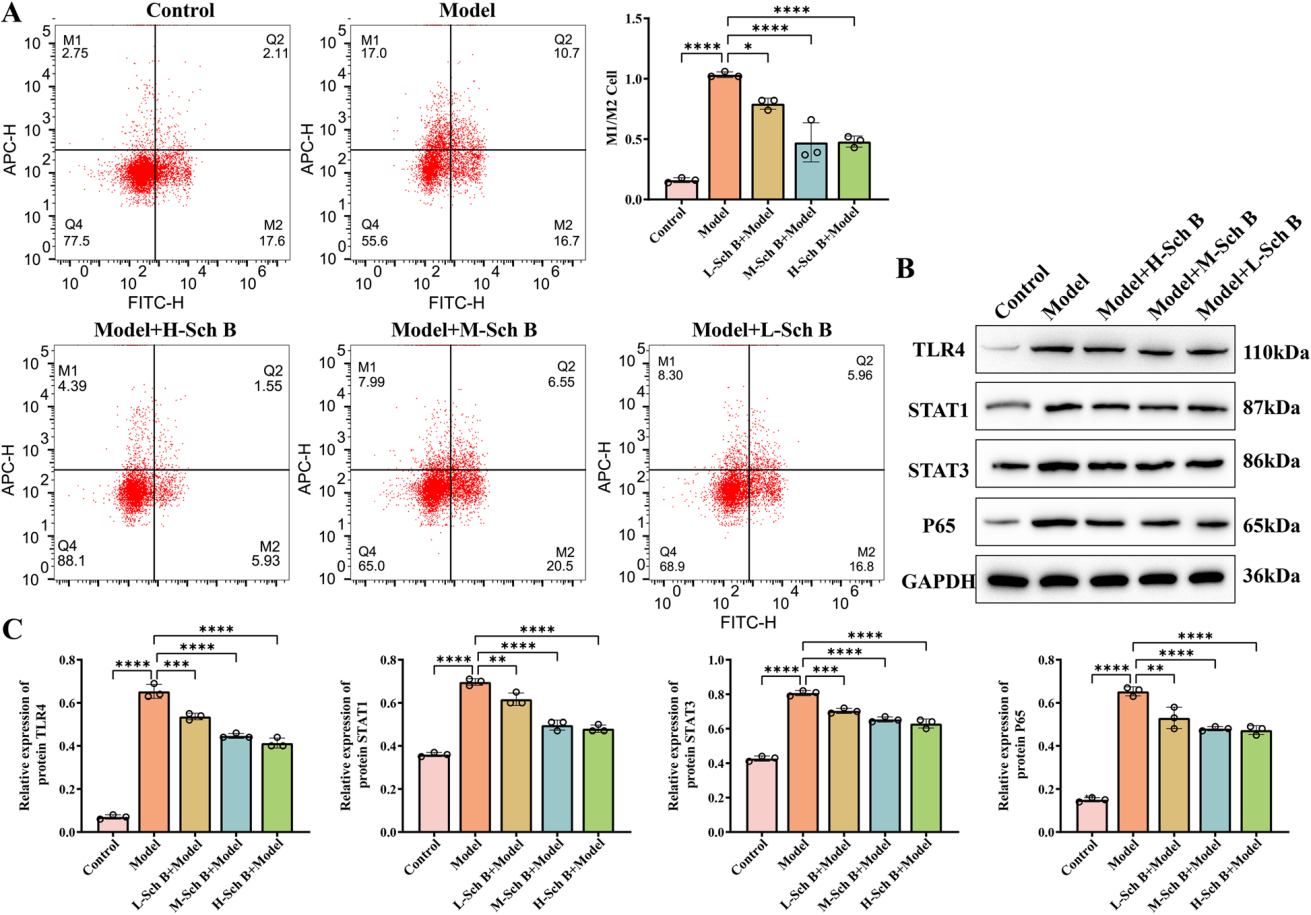
Effect of Sch B on Macrophages in COPD Rats. **A** M1/M2 macrophage ratio in rat lung tissues was detected by flow cytometry. **B** Protein expressions of TLR4, STAT1, STAT3, and P65 in lung tissues were detected by WB. All data are presented as mean ± SD. *N* = 3; *, *p* < 0.05; **, *p* < 0.01; ***, *p* < 0.001; ****, *p* < 0.0001

In terms of respiratory function, under the action of Sch B, FEV1 (*p* < 0.0001, Fig. 5A), FVC (*p* < 0.0001, Fig. 5B), tidal volume (*p* < 0.001, Fig. 5D), vital capacity (*p* < 0.0001, Fig. 5E), and minute ventilation volume (*p* < 0.0001, Fig. 5F) all increased with the increase in the dose of Sch B. Meanwhile, the respiratory rate (Fig. 5C) and inspiratory resistance (Fig. 5G) decreased (*p* < 0.0001). These results indicate that Sch B can effectively relieve the airflow limitation in rats with COPD. The results of arterial blood gas analysis showed that as the dose of Sch B increased, PaCO_2_ (*p* < 0.0001, Fig. 5H) decreased, while there was no significant change in PaO_2_ (Fig. 5F). This suggests that Sch B may have the effect of improving the gas exchange function of the lungs in COPD rats, yet its effect on improving the body’s oxygenation is not significant. After the intervention with the positive control drug, doxofylline tablets, the degree of lung tissue damage, body weight, body temperature, and the levels of inflammatory factors (IL-4, IL-6, IL-8, TNF-α) in the model rats all showed similar improvement trends as those with Sch B (*p* < 0.0001) (Fig. 4). The respiratory function indexes (FEV1, FVC, tidal volume, etc.), blood gas parameters (PaO_2_, PaCO_2_) were significantly optimized (*p* < 0.0001, Fig. 5A-I), and the intensity of its effect was comparable to that of the high-dose group of Sch B. This further verifies the therapeutic potential of Sch B for inflammation related to pulmonary fibrosis and respiratory dysfunction. Furthermore, flow cytometry analysis of the M1/M2 macrophage ratio in rat lung tissues revealed that with increasing doses of Schizandrin B, the M1/M2 macrophage ratio in the lung tissues of model rats exhibited a decreasing trend compared with the model group (*p* < 0.05, Fig. 6A). Western blot analysis at the protein level demonstrated that the protein expression levels of TLR4 (*p* < 0.001) and key molecules in the JAK-STAT signaling pathway, including STAT1 (*p* < 0.01), STAT3 (*p* < 0.001), and P65 (*p* < 0.01), all decreased in a dose-dependent manner with increasing doses of Schizandrin B (Fig. 6B-C). These results indicated that Schizandrin B exerts regulatory effects on the balance of macrophage polarization by inhibiting the TLR4 and JAK-STAT signaling pathways.

### COPD is ameliorated BySch B via regulation of the NF-κB/STAT1 signaling pathways and macrophage polarization through TLR4

**Fig. 7:**
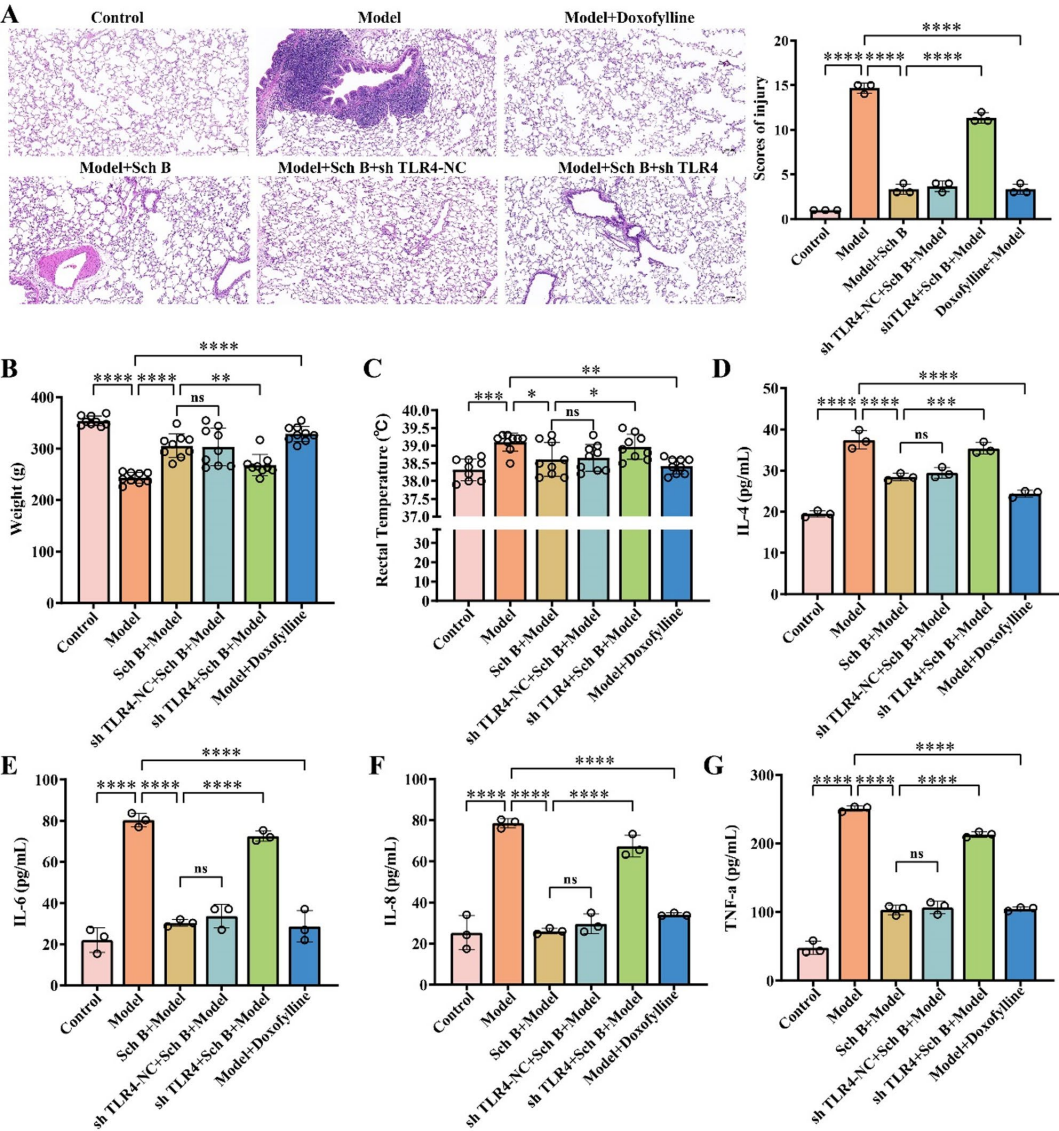
Effect of TLR4 Knockdown on Pathology and Inflammatory Factors in COPD Rats. **A** HE staining was performed on rat lung tissues (Scale bar: 200 μm). **B** Changes in rat body weight. **C** Rectal temperature measurement of rats. **D**-**G** Levels of IL-4, IL-6, IL-8, and TNF-α in rat bronchoalveolar lavage fluid were detected by ELISA. All data are presented as mean ± SD. *N* = 3; “*ns*”, no significant difference; *, *p* < 0.05; **, *p* < 0.01; ***, *p* < 0.001; ****, *p* < 0.0001

**Fig. 8:**
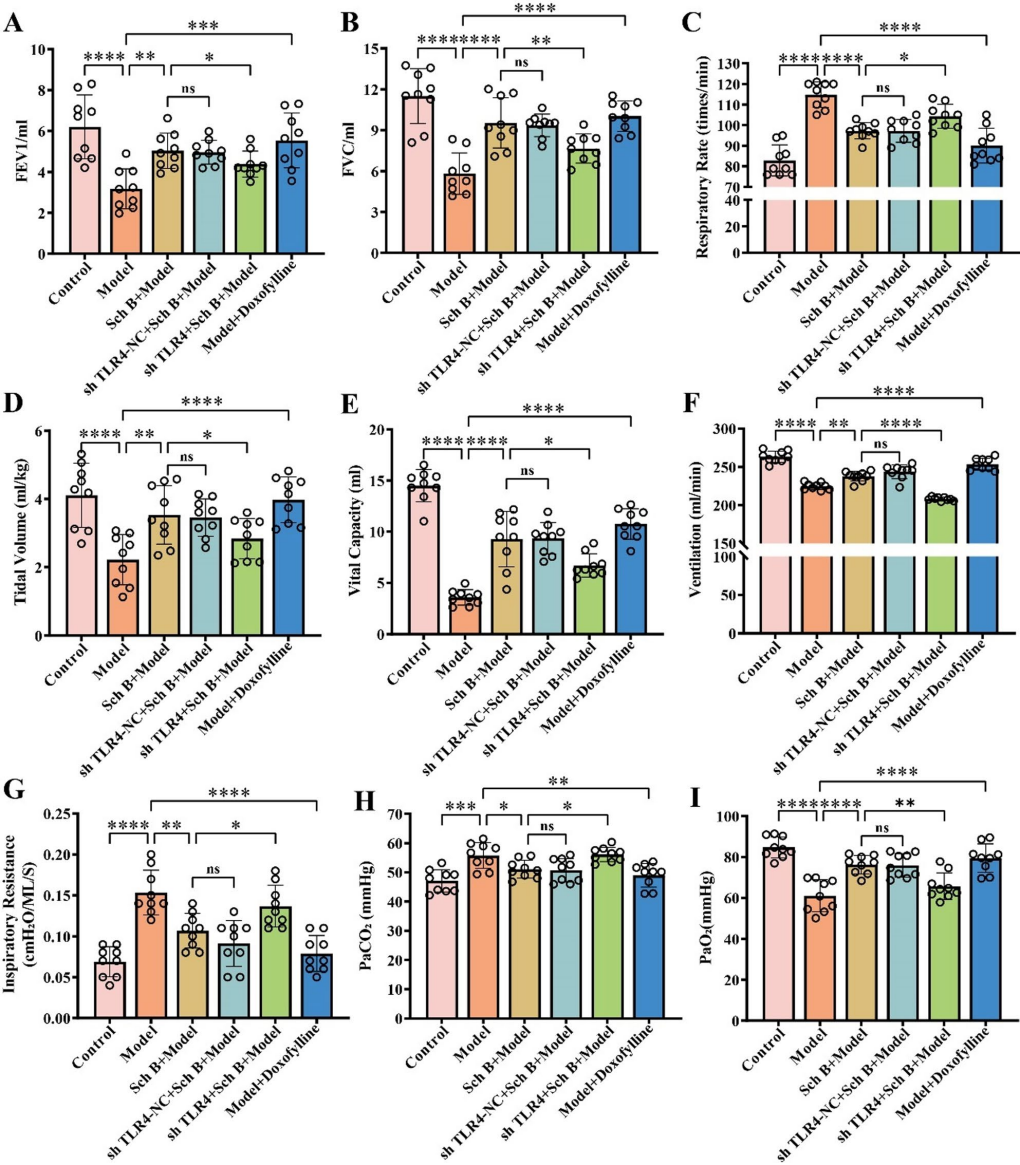
Effect of TLR4 Knockdown on Respiratory Function in COPD Rats. **A**-**G** Measurement of FEV1, FVC, respiratory rate, tidal volume, vital capacity, minute ventilation volume, and inspiratory resistance in rats. **H**-**I** Detection of PaCO_2_ and PaO_2_ in rats. All data are presented as mean ± SD. *N* = 3; “*ns*” represents no significant difference; *, *p* < 0.05; **, *p* < 0.01; ***, *p* < 0.001; ****, *p* < 0.0001

**Fig. 9:**
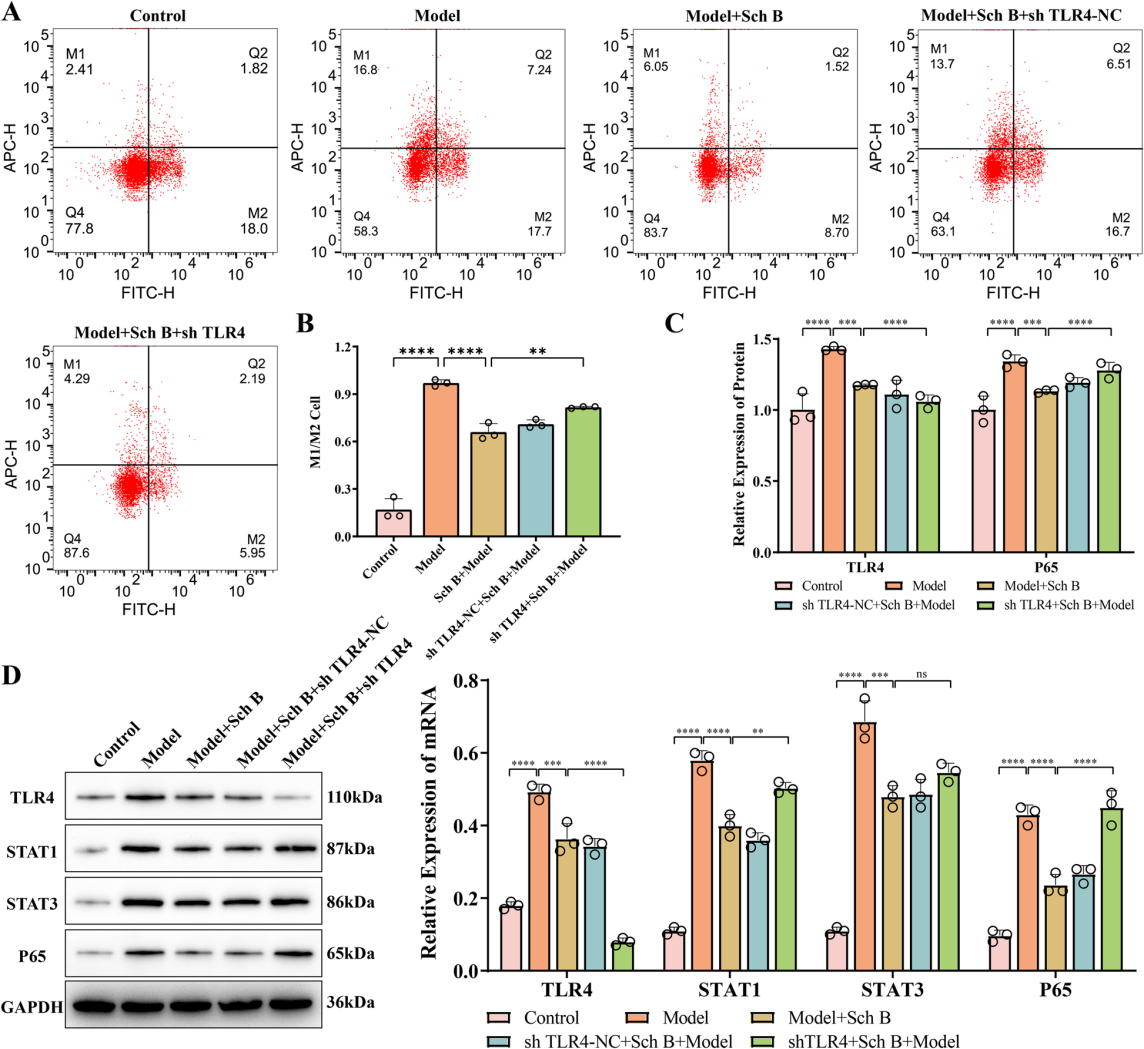
Effect of TLR4 Knockdown on Macrophages and the NF-κB/STAT1 Signaling Pathway in COPD rats. **A**-**B** M1/M2 macrophage ratio in rat lung tissues was detected by flow cytometry. **C** mRNA levels of TLR4 and P65 in lung tissues were detected by qPCR. **D** Protein expressions of TLR4, STAT1, STAT3, and P65 in lung tissues were detected by WB. All data are presented as mean ± SD. *N* = 3; “*ns*” represents no significant difference; **, *p* < 0.01; ***, *p* < 0.001; ****, *p* < 0.0001

To further investigate the mechanism underlying the role of TLR4 in the treatment of COPD by Sch B, high-dose Sch B intragastric administration combined with adeno-associated virus carrying TLR4 gene interference was performed in COPD model rats. HE staining results (Fig. 7A) showed that, the lung tissue structure was intact in the control group; severe alveolar wall disorder, widening, and thickening accompanied by massive inflammatory cell infiltration were observed in the Model group; pulmonary pathological damage was significantly alleviated in the Sch B + Model group (*p* < 0.0001). Compared with the Sch B + Model group, the degree of alveolar structure disorder, alveolar wall thickening, and inflammatory cell infiltration were significantly aggravated in the sh TLR4 + Sch B + Model group (*p* < 0.0001), suggesting that TLR4 knockdown could reverse the ameliorative effect of Sch B on pulmonary pathological damage in COPD rats. In addition, compared with the Sch B + Model group, the sh TLR4 + Sch B + Model group showed a significant decrease in rat body weight (*p* < 0.01), a significant increase in rectal temperature (*p* < 0.05), and markedly elevated levels of IL-4 (*p* < 0.001), IL-6 (*p* < 0.0001), IL-8 (*p* < 0.0001), and TNF-α (*p* < 0.0001) in bronchoalveolar lavage fluid (Fig. 7B-G). Pulmonary function test results demonstrated that TLR4 knockdown reversed Sch B-mediated improvement in pulmonary ventilation function, forced FEV1 (*p* < 0.05, Fig. 8A), FVC (*p* < 0.01, Fig. 8B), tidal volume (*p* < 0.05, Fig. 8D), vital capacity (*p* < 0.05, Fig. 8E), and minute ventilation volume (*p* < 0.0001, Fig. 8F) were all significantly decreased, while respiratory rate (*p* < 0.05, Fig. 8C) and inspiratory resistance (*p* < 0.05, Fig. 8G) were significantly increased. These findings suggested that TLR4 knockdown could antagonize the regulatory effects of Sch B on body weight and temperature, and reverse its inhibitory effect on pulmonary pro-inflammatory factors in COPD rats. Arterial blood gas analysis showed that the sh TLR4 + Sch B + Model group had a significant increase in PaCO2 (*p* < 0.05, Fig. 8H) and a significant decrease in PaO2 (*p* < 0.01, Fig. 8I). This indicated that TLR4 knockdown could impair the ameliorative effect of Sch B on pulmonary gas exchange function and exacerbate hypoxemia and hypercapnia. Flow cytometry analysis of the M1/M2 macrophage ratio in rat lung tissues revealed that compared with the Sch B + Model group, the M1/M2 macrophage ratio was significantly increased in the sh TLR4 + Sch B + Model group (*p* < 0.01, Fig. 9A-B). Molecular level detection results showed that: compared with the Sch B + Model group, the sh TLR4 + Sch B + Model group exhibited significantly decreased mRNA and protein expression levels of TLR4, while the mRNA and protein expression levels of P65 were significantly increased (*p* < 0.0001, Fig. 9C-D); meanwhile, the protein level of STAT1 was significantly elevated in this group (*p* < 0.01), whereas no significant change was observed in the protein level of STAT3 (*p* > 0.5, Fig. 9C-D).

## Discussion

COPD is one of the leading causes of morbidity, mortality, as well as consumption of healthcare resources globally, having been recognized as a major global healthcare burden [23]. In this study, a rat model of COPD was successfully established by combining LPS with cigarette smoke exposure. This model exhibited pathological characteristics similar to those of COPD in terms of pathological changes in lung tissues, overall physical state, pulmonary function, blood gas indexes, and levels of inflammatory factors. These findings validated the effectiveness of the model establishment [24].

As a basic formula for clinically treating lung qi deficiency and lung-kidney qi deficiency, Bufei Decoction has been proven to treat COPD through multiple mechanisms, such as regulating inflammatory responses and correcting immune imbalance [25, 26]. However, existing studies mostly remain at the level of the overall efficacy of the compound prescription, lacking systematic correlation verification of the “compound prescription-monomer-target-pathway” axis. Taking Bufei Decoction as the entry point, this study combined network pharmacology, focused on its core active component Sch B, and thoroughly explored the key targets and regulatory networks underlying its therapeutic effect on COPD. This research achieves precise extension from the overall efficacy of the compound prescription to the molecular mechanism of the monomer. The results of this study confirmed that Bufei Decoction could increase body weight, reduce body temperature, improve lung function indices, and regulate blood gas parameters in COPD model rats, laying a foundation for subsequent studies on monomer components.Network pharmacology analysis showed that six core components of Bufei Decoction, including *Schisandra chinensis*, *Astragalus membranaceus*, and *Morus alba L. bark*, synergistically exerted the effects of tonifying the lung and kidney, replenishing qi, relieving cough, and relieving asthma. Its therapeutic targets were involved in biological processes such as lipopolysaccharide-mediated signaling pathways and macrophage activation, reflecting the advantage of traditional Chinese medicine compounds in “multi-component, multi-target, and multi-pathway” [18]. Furthermore, this study identified Sch B as the core active monomer, providing new evidence for clarifying the material basis of the compound’s therapeutic efficacy.

Sch B is the main active component of *Schisandra chinensis*. Previous studies have confirmed that it possesses multiple pharmacological activities, including anti-tumor, anti-oxidant, and anti-inflammatory effects [27], but the core mechanism underlying its regulation of immune cell function has not been thoroughly explored. This study demonstrated that Sch B significantly alleviated lung inflammation in COPD model rats, reduced the levels of inflammatory factors (IL-1β, IL-6, TNF-α) in BALF, and effectively improved respiratory function and blood oxygen saturation in rats. Previous studies have also confirmed that Sch B can reduce pyroptosis by inhibiting the NOD-like receptor protein 3 (NLRP3) inflammasome, thereby alleviating airway inflammation and remodeling in asthmatic rats [28]. Additionally, it can attenuate high glucose-induced damage to vascular endothelial cells by regulating the Noxa/Hsp27/NF-κB signaling pathway, further mitigating inflammatory responses [29]. Combined with its therapeutic efficacy comparable to that of the positive control drug doxofylline, these findings further confirm the therapeutic potential of Sch B in COPD model rats.

More importantly, for the first time, this study clarified that Sch B improves COPD pathological damage by regulating the balance of macrophage polarization. In the pathological process of COPD, abnormal macrophage polarization has been confirmed as a key factor driving inflammatory responses and lung tissue damage [30]. This study verified that Sch B could dose-dependently reduce the M1/M2 macrophage ratio in lung tissue, thereby effectively regulating the state of macrophage polarization. This mechanism of action has also been supported by studies in other disease models. For example, in a liver fibrosis model, Sch B can inhibit macrophage activation and polarization through the PPARγ-mediated NF-κB signaling pathway [31]. Based on these findings, it is speculated that the inhibitory effect of Sch B on the NF-κB and JAK-STAT signaling pathways may be one of the core mechanisms underlying its regulatory function in macrophage polarization. The results of this study showed that Sch B significantly downregulated the expression levels of P65, a key transcription factor in the NF-κB pathway, as well as STAT1 and STAT3, key molecules in the JAK-STAT pathway. As a core pathway regulating the expression of pro-inflammatory genes such as cytokines and chemokines, NF-κB plays a crucial role in the survival, activation, and differentiation of innate immune cells and inflammatory T cells [32]. Studies by Alanazi et al. have confirmed that NF-κB activity is significantly enhanced in the lung tissue of COPD patients, accompanied by inflammatory cell infiltration and high expression of pro-inflammatory factors such as TNF-α, IL-6, and IL-8.Notably, NF-κB inhibitors can significantly inhibit the above pathological processes [33], which is consistent with the results of this study.In addition, the JAK-STAT signaling pathway is widely involved in the signal transduction of cytokines and growth factors, and exerts important effects on inflammatory responses, immune function regulation, and tissue repair [34, 35]. Studies by Irey et al. have indicated that inhibition of STAT1 can attenuate M1 macrophage polarization, while inhibition of STAT3 can weaken M2 macrophage polarization [36]. Moreover, the expression levels of key proteins in the JAK-STAT pathway and pro-inflammatory factors such as IL-6 and TNF-α are significantly increased in the lung tissue of COPD patients ) [37–39]. This is highly consistent with the results of this study that Sch B downregulates the expression of STAT1 and STAT3, further confirming the role of this pathway in Sch B-mediated regulation of macrophage polarization.

Network pharmacology analysis further revealed that TLR4 is the core key target of Bufei Decoction in intervening COPD. This predicted result was highly consistent with the molecular docking experimental findings—the binding energy between Sch B and TLR4 protein reached − 7.7 kJ/mol. According to the conventional evaluation criteria for molecular docking studies, a binding energy ≤ -5 kJ/mol indicates good binding activity between ligand and target [40]. The binding energy of -7.7 kJ/mol was significantly lower than this threshold, demonstrating high binding specificity and affinity between Sch B and TLR4 protein.This provides a reliable molecular basis for the direct interaction between them. After TLR4 gene knockdown in COPD model rats, the therapeutic effect of Sch B was significantly reversed. Not only were the core therapeutic effects of Sch B—improving lung function and alleviating lung inflammation—completely abolished, but its inhibitory effect on the NF-κB and JAK-STAT signaling pathways was also completely blocked. This ultimately led to an increased M1/M2 macrophage ratio, failure of macrophage polarization regulation, and further exacerbation of inflammatory infiltration and respiratory dysfunction in COPD model rats. As a core regulatory molecule in innate immune responses, TLR4 can activate the NF-κB signaling pathway through the myeloid differentiation factor 88 (MyD88)-dependent pathway.This activation drives the massive release of pro-inflammatory factors such as IL-1β, IL-6, and TNF-α [41]. Meanwhile, TLR4 can also promote endothelial-mesenchymal transition by regulating extracellular matrix metabolism and intercellular communication. It participates in pulmonary vascular remodeling and inflammatory cascade amplification, thereby exacerbating lung tissue damage in COPD [42–44]. Studies by Sidletskaya et al. have also confirmed that TLR4 occupies a key hub position in the COPD inflammatory network. It is directly involved in core pathological processes such as fibrosis, emphysema, and lung function decline [45], providing important pathological evidence for the target selection in this study. In addition, studies on ischemic stroke models have verified that the activated TLR4/NF-κB pathway can further activate the JAK-STAT pathway in a cascading manner, aggravating tissue inflammatory responses [46]. The results of this study are consistent with these findings, indicating that the TLR4/NF-κB/JAK-STAT cascade regulatory mode may be a common cross-disease inflammatory regulatory mechanism. For the first time, this study extends this mechanism to the field of COPD and clarifies that Sch B can block this pathway cascade by targeting TLR4.

### Limitations

Although this study has clarified the core mechanism and key targets of Sch B in intervening COPD, certain limitations remain and need to be further addressed in subsequent research. Firstly, the sample size of animal experiments was relatively small. Although a mature COPD rat model induced by lipopolysaccharide combined with cigarette smoke was used in this study, and the experimental data showed significant statistical differences, the small sample size may compromise statistical power and result reproducibility. Future studies can be conducted by expanding the sample size, adding independent duplicate experimental cohorts, or performing multi-center animal experiments to further verify the stability and reliability of the core conclusions. Secondly, the depth of mechanism exploration needs to be enhanced. This study confirmed that Sch B regulates macrophage polarization through the TLR4/NF-κB/JAK-STAT pathway, but the molecular details of this regulatory network remain to be further elucidated. On one hand, although molecular docking experiments confirmed high-affinity binding between Sch B and TLR4, verification of TLR4 binding site mutations and complex structure modeling analysis are lacking, making it impossible to clarify the key amino acid residues and conformational changes involved in their binding. On the other hand, the crosstalk between the NF-κB and JAK-STAT pathways has not been thoroughly explored. In subsequent studies, TLR4 site-directed mutagenesis and surface plasmon resonance technology can be used to verify binding kinetic characteristics, and ChIP-seq and other technologies can be combined to analyze the downstream transcriptional regulatory network of the pathway, thereby further improving the depth and accuracy of mechanism research.

## Conclusions

This study reveals that Bufei Decoction exerts its therapeutic effect on COPD through the synergistic action of multiple components. In particular, its core active component, Sch B, targets TLR4, regulates the NF-κB and JAK-STAT signaling pathways, influences macrophage polarization and the inflammatory response. This finding provides new insights for the development of COPD treatment strategies based on Sch B or TLR4-targeted drugs.

## Supplementary Information

Supplementary Material 1.

## Data Availability

No datasets were generated or analysed during the current study.

## Abbreviations

COPD: Chronic obstructive pulmonary disease
TLR4: toll-like receptor 4
JAK1: Janus kinase 1
STAT3: Transducer and activator of transcription 3
NF-κB: the nuclear factor-kappa B
WHO: World Health Organization
IFN-γ: SInterferon-γ
LPS: Lipopolysaccharide
ROS: Reactive oxygen species
MAPK: Mitogen-activated protein kinase
PI3K-Akt: Phosphatidylinositol 3-kinase-Akt
JAK-STAT: Janus kinase-signal transducer and activator of transcription
TCM: Traditional Chinese medicine
HE: Hematoxylin and eosin
BALF: bronchoalveolar lavage fluid
ELISA: Enzyme-linked immunosorbent assay
EP: Eppendorf
IL-1β: Interleukin-1 beta
IL-4: Interleukin-4
IL-6: Rat interleukin-6
IL-8: Interleukin-8
IL-10: Interleukin-10
IL-13: Interleukin-13
TNF-α: Tumor necrosis factor-alpha
TCMSP: Traditional Chinese Medicine Systems Pharmacology Database and Analysis Platform
PPI: Construction of the protein-protein interaction
RCSB: Research Collaboratory for Structural Bioinformatics
GO: Gene Ontology
KEGG: Kyoto Encyclopedia of Genes and Genomes
shRNA: specific short-hairpin RNA
AAV: Deno-associated virus
PBS: Phosphate-buffered saline
RT-qPCR: 2.17 Real-time quantitative polymerase chain reaction
GAPDH: Glyceraldehyde − 3 - phosphate dehydrogenase
iNOS: Inducible nitric oxide synthas
CD86: Cluster of differentiation 86
Arg1: Arginase 1
CD206: Cluster of differentiation 206
PMSF: phenylmethylsulfonyl fluoride
ECL: Enhanced chemiluminescence
FEV1: Forced expiratory volume in one second
PaCO_2_: the partial pressure of carbon dioxide in arterial blood
PaO2: the partial pressure of oxygen in arterial blood
FVC: forced vital capacity
CASP3: Aspase 3
NFKB1: Nuclear factor kappa B subunit 1
JUN: Jun proto-oncogene
TNF: Tumor necrosis factor
BCL2: B-cell lymphoma 2
PTGS2: Prostaglandin G/H synthase 2
BP: Biological Processes
CC: Cellular Components
MF: Molecular Functions
NOD-NLRP3: Nucleotide-binding oligomerization domain-like receptor protein 3
MyD88: Myeloid differentiation primary response 88
