# Supplementary figures and images for: Investigating the therapeutic mechanism of Bufei Decoction in COPD: Schisandrin B targets the TLR4/NF-κB/JAK-STAT signaling pathway
Source: Hereditas. 2025 Dec 27;163:17. doi: 10.1186/s41065-025-00629-8 (PMC12853953; doi:10.1186/s41065-025-00629-8)

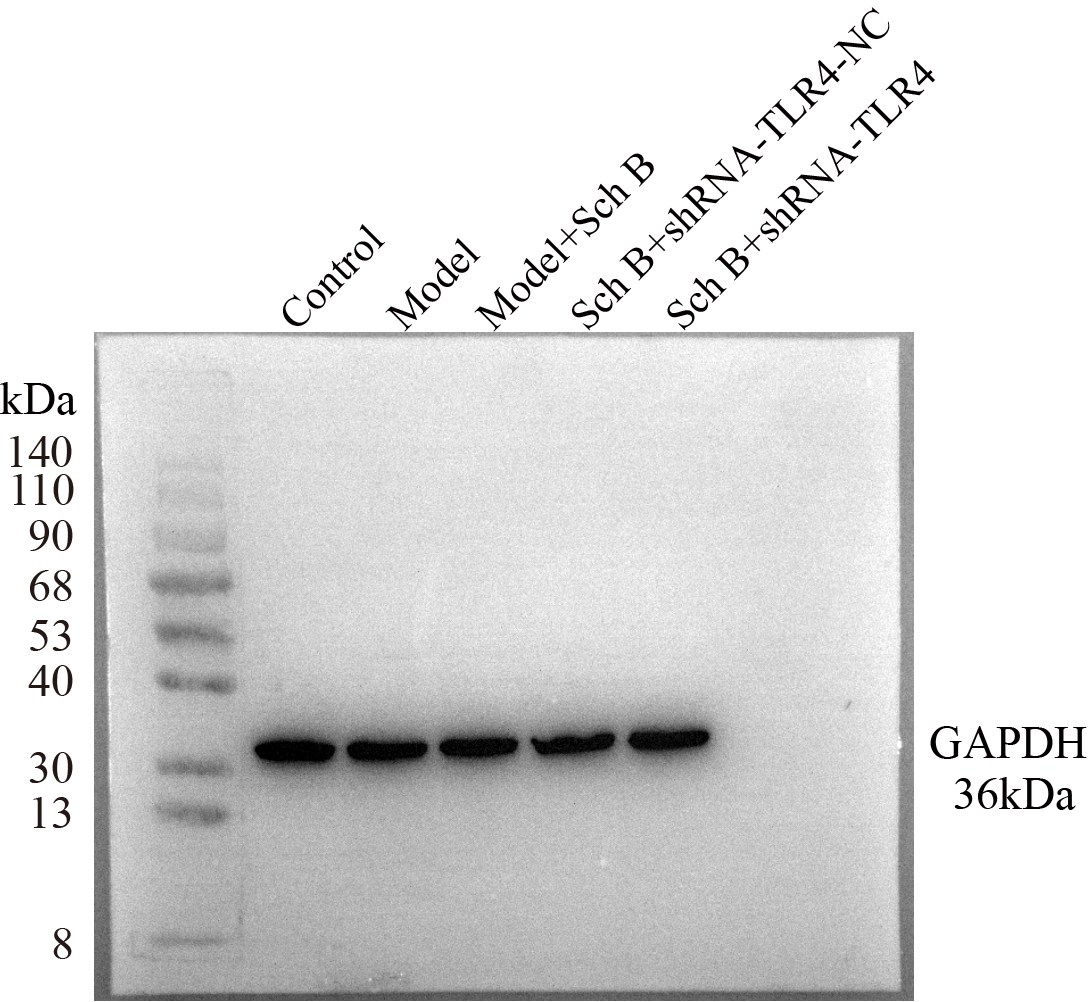

Supplement: Supplementary file 1 — Supplementary Material 1. [file 41065_2025_629_MOESM1_ESM.zip › Figure 7/GADPH.jpg]

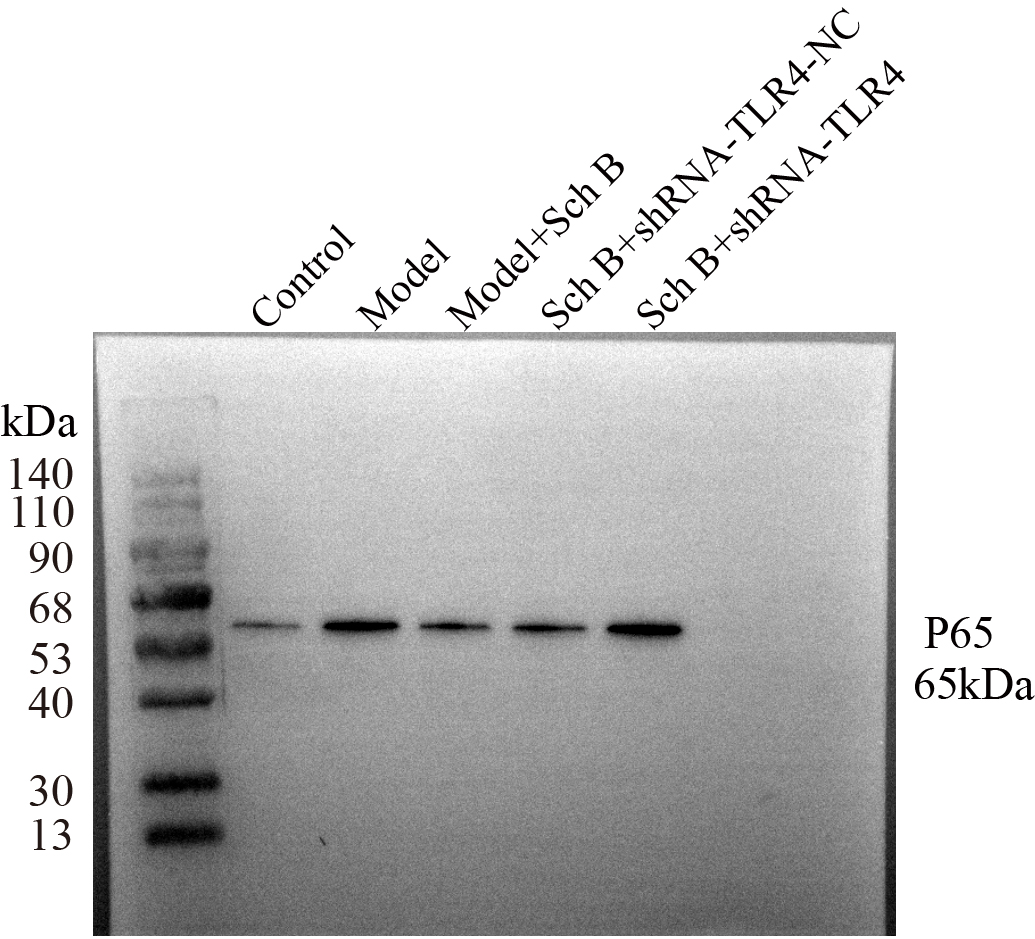

Supplement: Supplementary file 1 — Supplementary Material 1. [file 41065_2025_629_MOESM1_ESM.zip › Figure 7/P65.jpg]

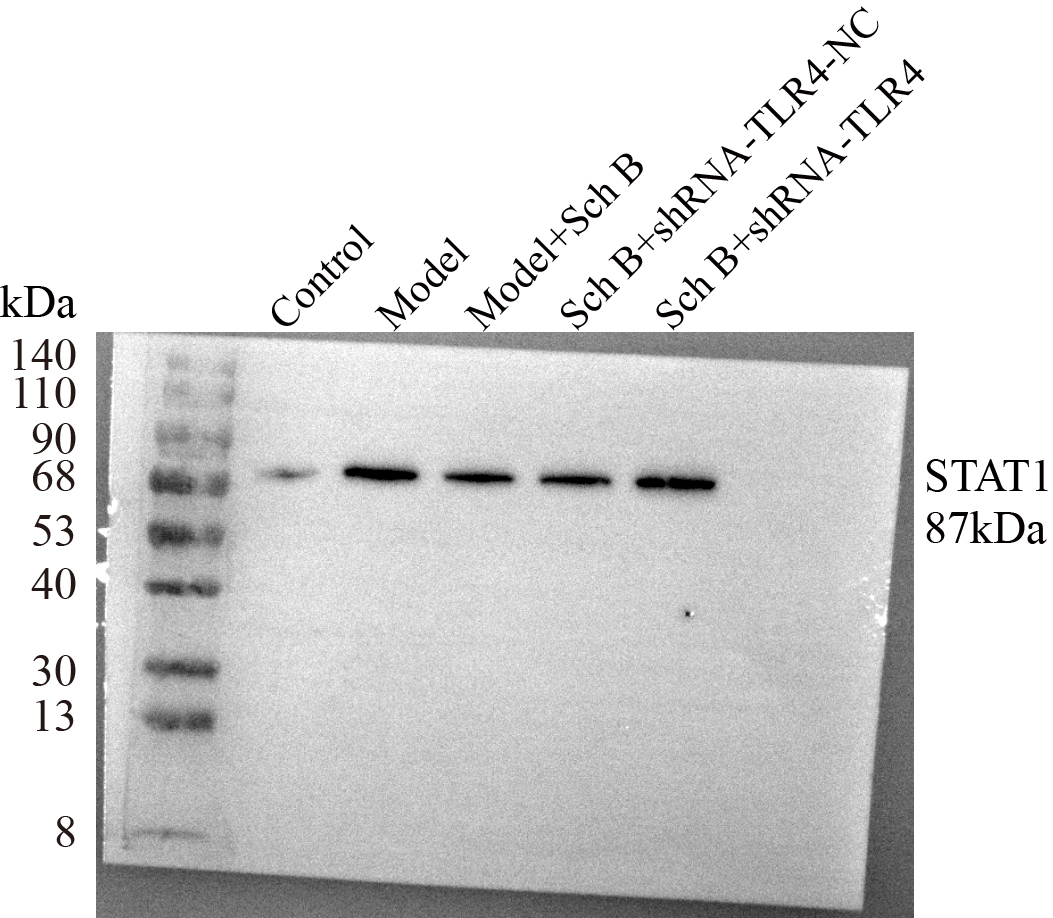

Supplement: Supplementary file 1 — Supplementary Material 1. [file 41065_2025_629_MOESM1_ESM.zip › Figure 7/STAT1.jpg]

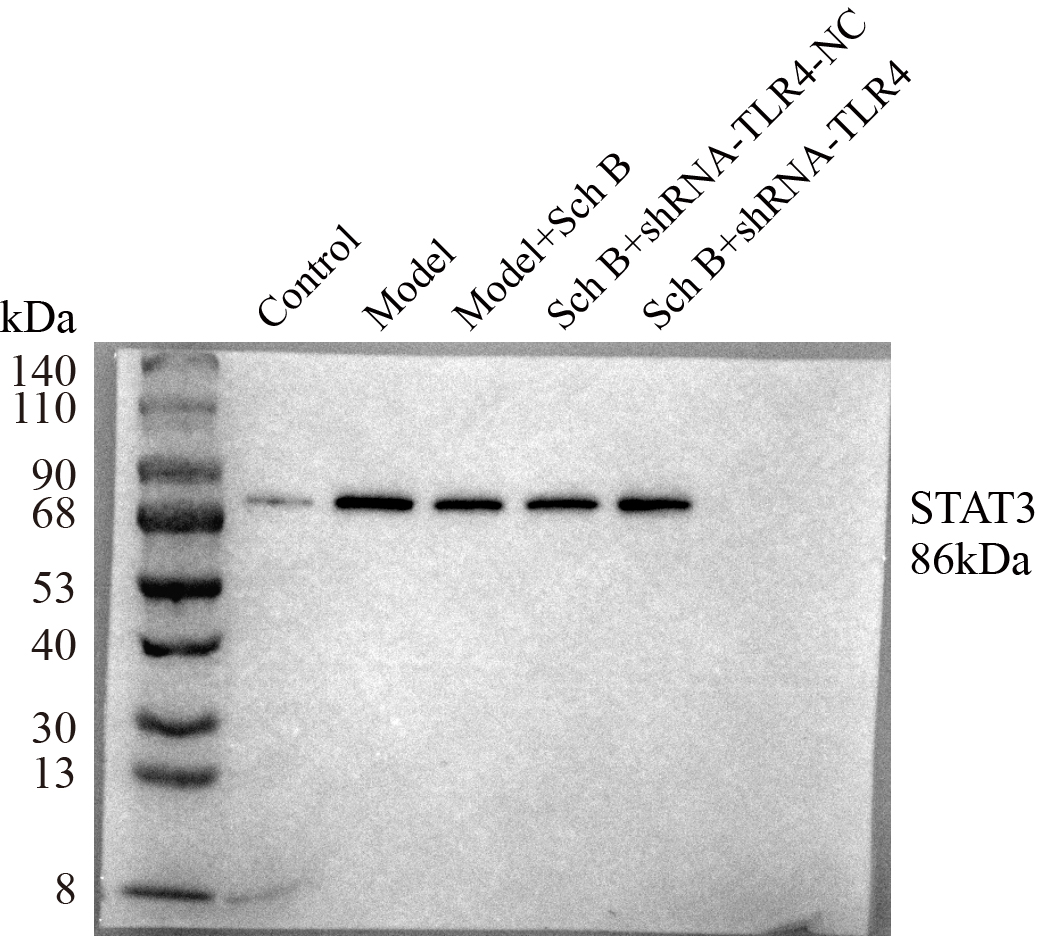

Supplement: Supplementary file 1 — Supplementary Material 1. [file 41065_2025_629_MOESM1_ESM.zip › Figure 7/STAT3.jpg]

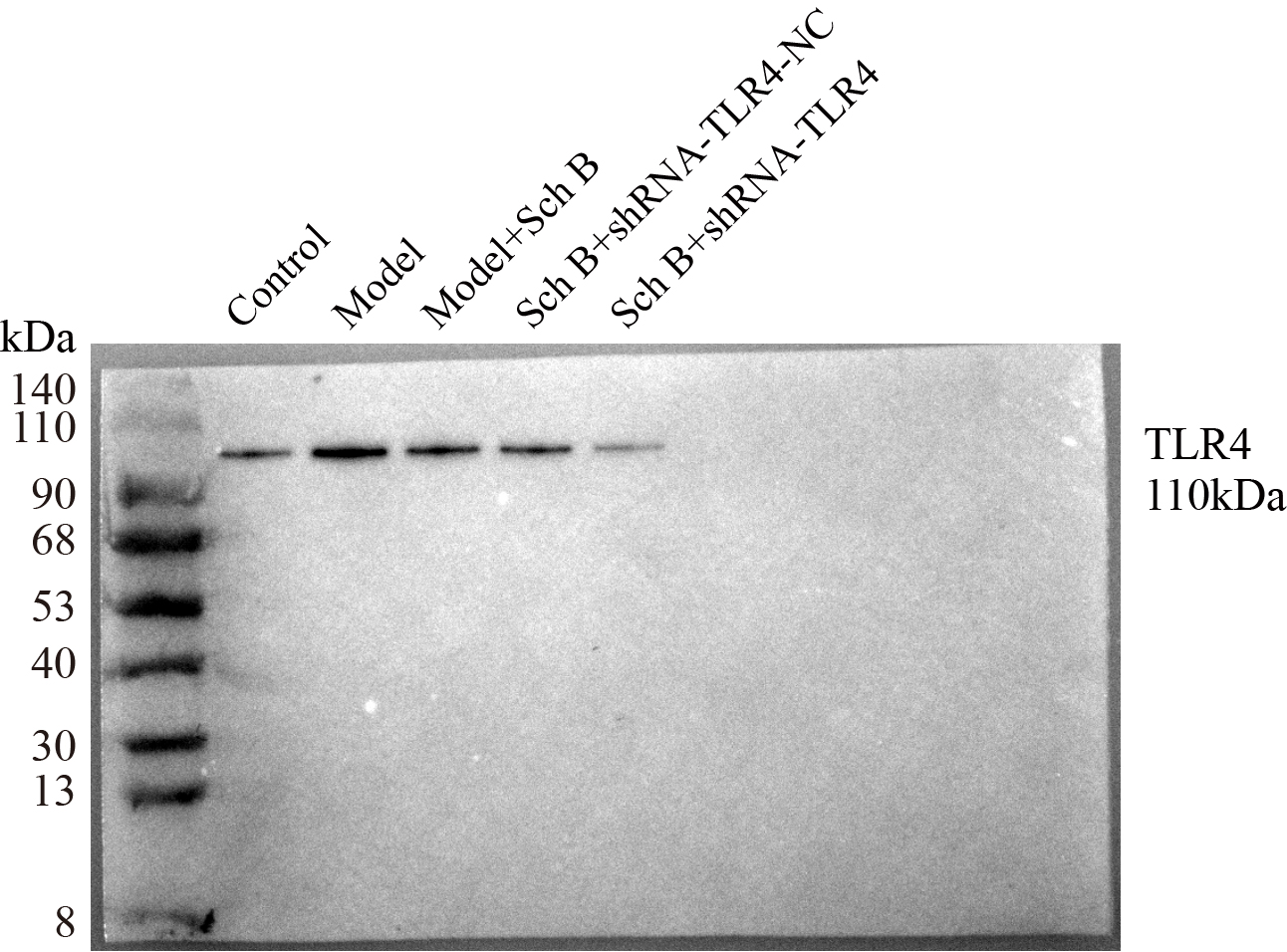

Supplement: Supplementary file 1 — Supplementary Material 1. [file 41065_2025_629_MOESM1_ESM.zip › Figure 7/TLR4.jpg]

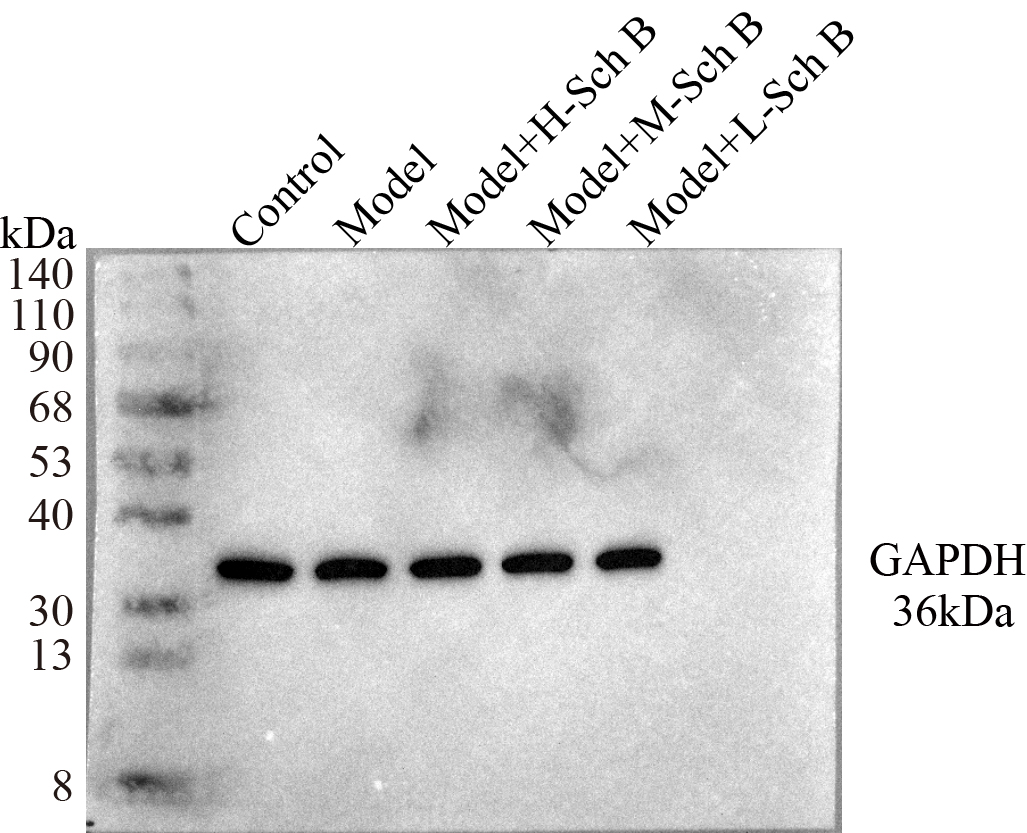

Supplement: Supplementary file 1 — Supplementary Material 1. [file 41065_2025_629_MOESM1_ESM.zip › Figure 6/GAPDH.jpg]

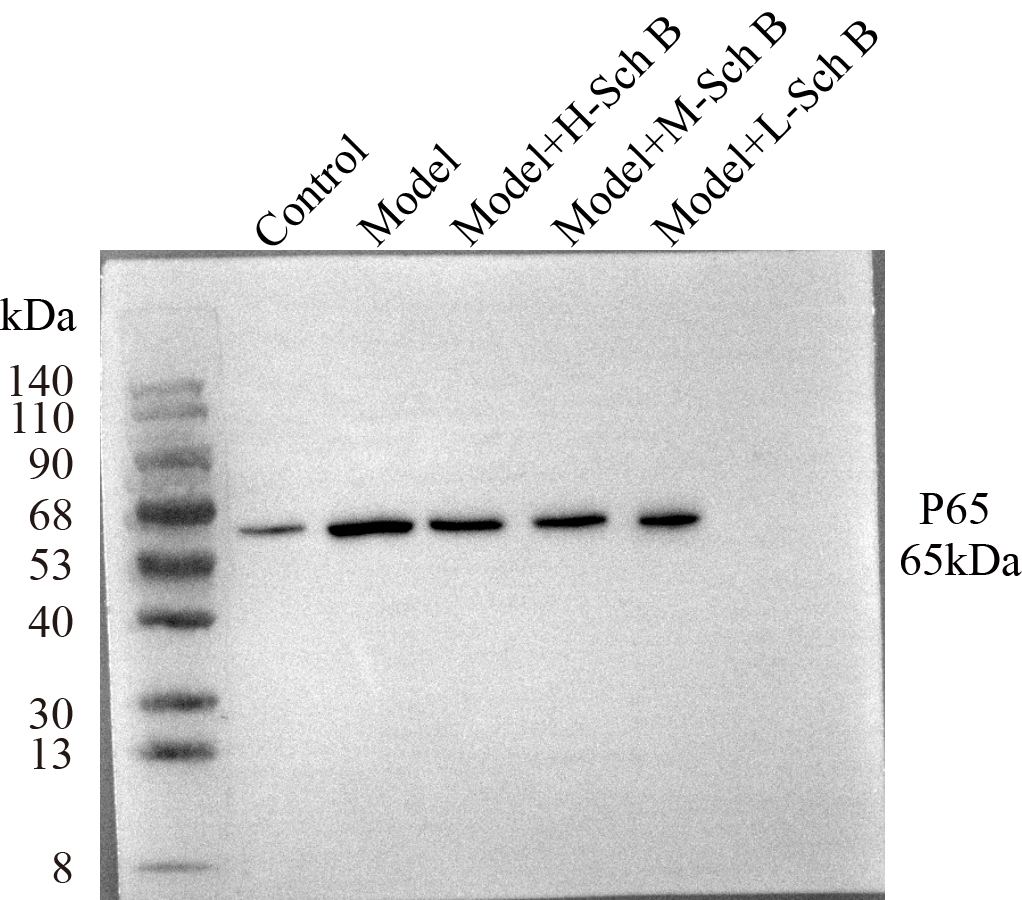

Supplement: Supplementary file 1 — Supplementary Material 1. [file 41065_2025_629_MOESM1_ESM.zip › Figure 6/P65.jpg]

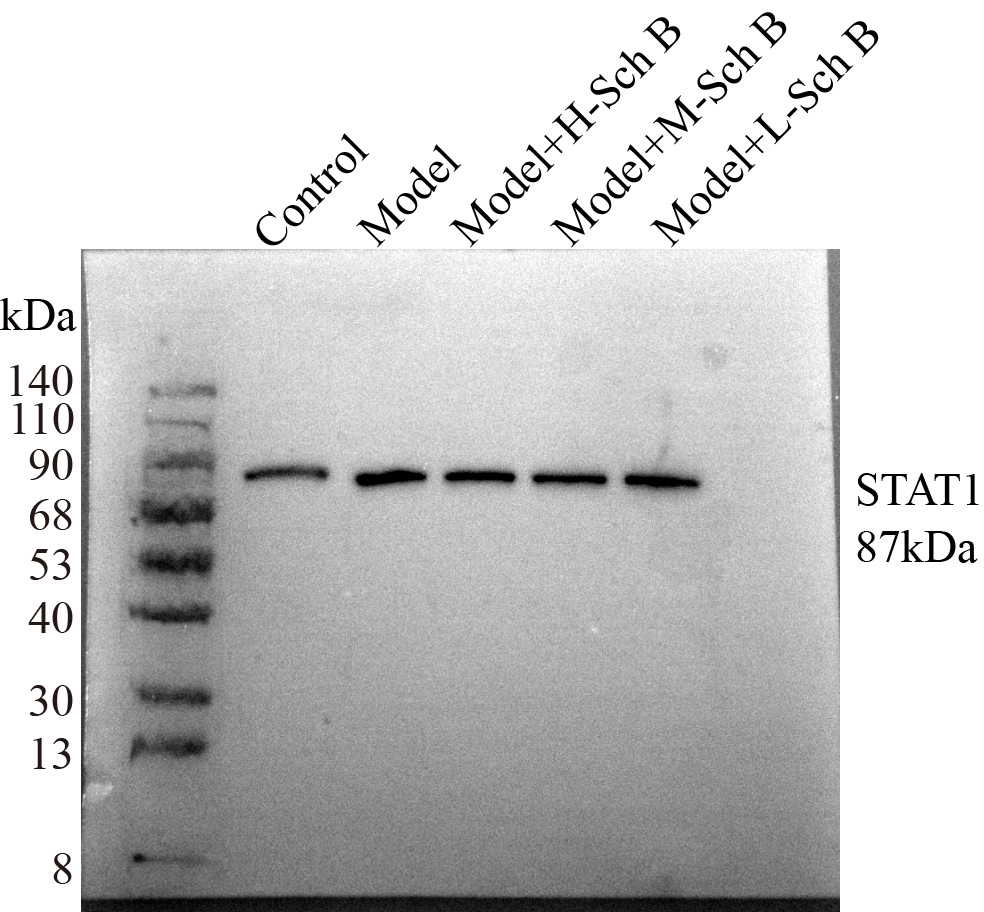

Supplement: Supplementary file 1 — Supplementary Material 1. [file 41065_2025_629_MOESM1_ESM.zip › Figure 6/STAT1.jpg]

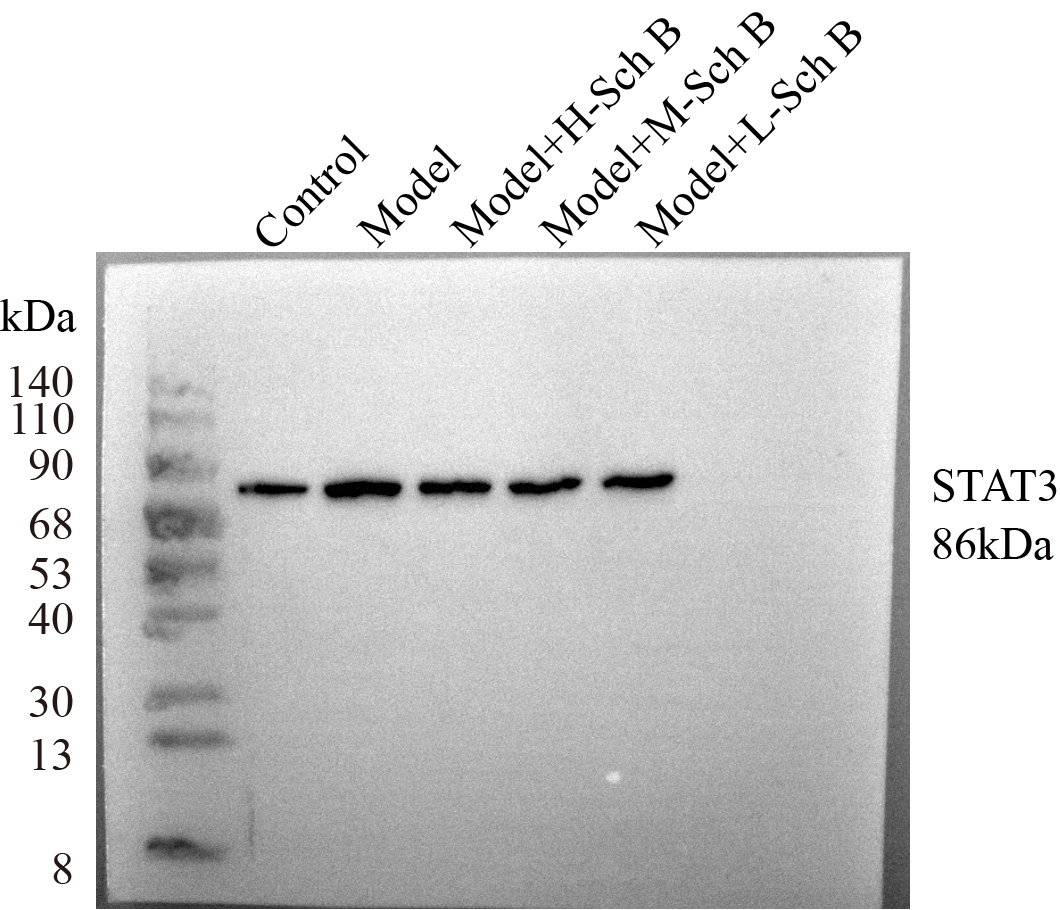

Supplement: Supplementary file 1 — Supplementary Material 1. [file 41065_2025_629_MOESM1_ESM.zip › Figure 6/STAT3.jpg]

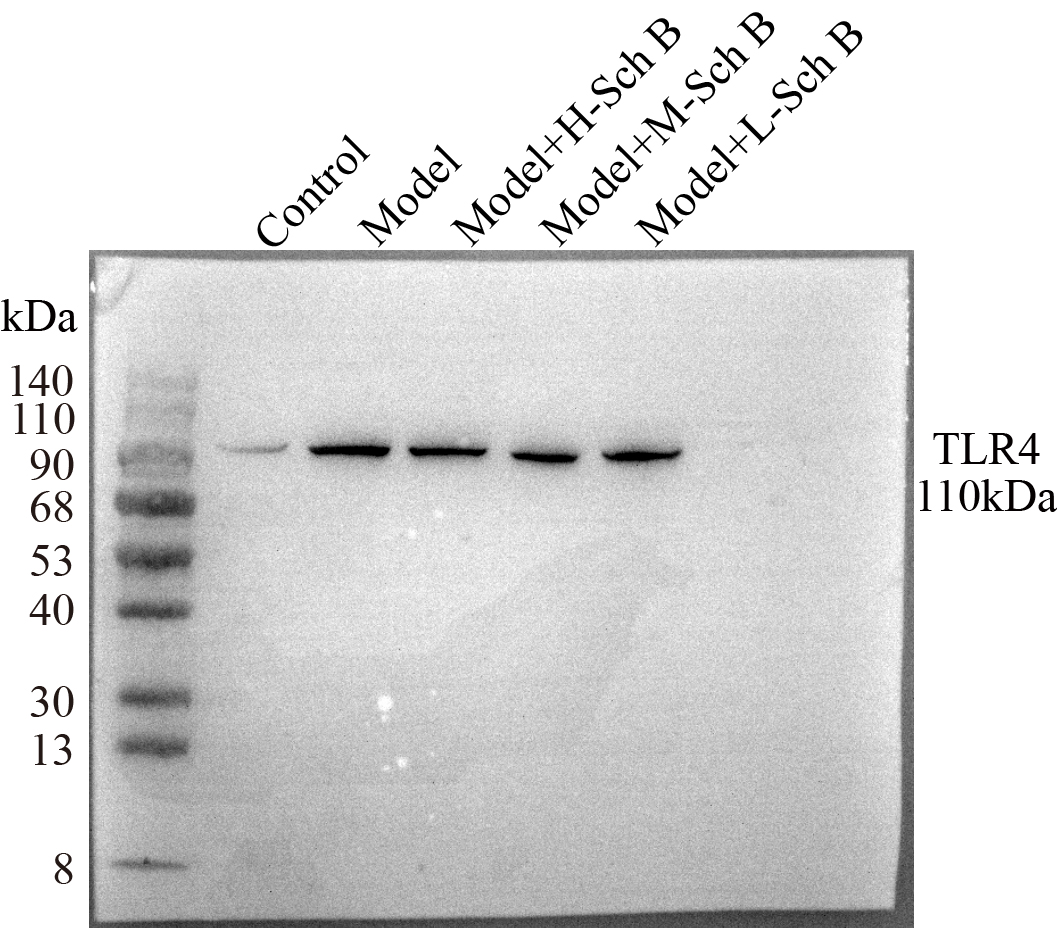

Supplement: Supplementary file 1 — Supplementary Material 1. [file 41065_2025_629_MOESM1_ESM.zip › Figure 6/TLR4.jpg]
